# Caste-specific expressions and diverse roles of *takeout* genes in the termite *Reticulitermes speratus*

**DOI:** 10.1038/s41598-023-35524-7

**Published:** 2023-05-24

**Authors:** Kokuto Fujiwara, Akimi Karasawa, Takumi Hanada, Mutsuaki Tobo, Tousuke Kaneko, Mizuna Usui, Kiyoto Maekawa

**Affiliations:** 1 Graduate School of Science and Engineering, University of Toyama, Gofuku, Toyama, 930-8555 Japan; 2 Department of Biology, Faculty of Science, University of Toyama, Gofuku, Toyama, 930-8555 Japan; 3 Faculty of Science, Academic Assembly, University of Toyama, Gofuku, Toyama, 930-8555 Japan

**Keywords:** Evolutionary developmental biology, Social evolution

## Abstract

Acquisition of novel functions caused by gene duplication may be important for termite social evolution. To clarify this possibility, additional evidence is needed. An important example is *takeout*, encoding juvenile hormone binding protein. We identified 25 *takeout*s in the termite *Reticulitermes speratus* genome. RNA-seq revealed that many genes were highly expressed in specific castes. Two novel paralogs (*RsTO1*, *RsTO2*) were tandemly aligned in the same scaffold. Real-time qPCR indicated that *RsTO1* and *RsTO2* were highly expressed in queens and soldiers, respectively. Moreover, the highest *RsTO1* expression was observed in alates during queen formation. These patterns were different from *vitellogenins*, encoding egg-yolk precursors, which were highly expressed in queens than alates. In situ hybridization showed that *RsTO1* mRNA was localized in the alate-frontal gland, indicating that RsTO1 binds with secretions probably used for the defence during swarming flight. In contrast, increased *RsTO2* expression was observed approximately 1 week after soldier differentiation. Expression patterns of *geranylgeranyl diphosphate synthase*, whose product functions in the terpenoid synthesis, were similar to *RsTO2* expression. In situ hybridization indicated *RsTO2*-specific mRNA signals in the soldier-frontal gland. RsTO2 may interact with terpenoids, with a soldier-specific defensive function. It may provide additional evidence for functionalization after gene duplication in termites.

## Introduction

Termites are eusocial cockroaches^1,2^ that exist as morphologically distinct castes within the same colony^3,4^. Termite castes (reproductives, soldiers, and workers) are good examples of polyphenism; all individuals in a colony have essentially the same genome^5,6^. Termite genomes have been sequenced in some species, and many caste-specific genes have been identified (e.g.,^7–9^). These genes include those involved in caste-specific morphologies and division of labor^10,11^. Interestingly, the number of soldier-specific expressed genes tends to be larger than those of any other caste^10,11^. Clarifying the evolutionary processes and functions of these genes is crucial for understanding the proximate factors underlying termite caste polyphenism.

Recently, a genome-based study in the subterranean termite *Reticulitermes speratus* indicated that caste-specific expressed genes were mostly derived from multi-gene families and that gene duplication probably promoted the evolution of such paralogous genes^9^. Based on next-generation sequencing technologies, caste-specific expression patterns were clarified in each paralog of some genes involved in social life (e.g., *lipocalins* and *beta-glucosidases*). Moreover, the genomic position and cDNA sequences were identified in four paralogous genes of vitellogenin (Vg; the major yolk protein precursor), and caste-specific expression patterns were verified by real-time quantitative PCR (qPCR)^12^. Further accumulation of data on such multi-gene family genes is required for a general understanding of their roles in termite social evolution.

Another important example of a multi-gene family is the juvenile hormone binding protein (JHBP) superfamily *takeout*. Generally, in insects, there are many *takeout* genes in the genome, and paralog-specific expression has been identified in some species (e.g.,^13^). JH is a central factor regulating termite caste differentiation^14^, and *takeout* genes normally contain the conserved JHBP domain. Originally, *takeout* was identified in the circadian output pathway and starvation response in *Drosophila melanogaster*^15,16^. However, in the cockroach *Diploptera punctata*, takeout is involved in JH and/or JH precursor transport^16^. In the honeybee *Apis mellifera*, *takeout* (*GB19811*) expression patterns in workers might be affected by JH titer changes and may be involved in JH-mediated regulation of development^17,18^. Moreover, transcript initiation of *moling*, the homolog of *takeout*, is caused by nutritional intake and a decrease in JH titer in the moth *Manduca sexta*^18^. In termites, diverse *takeout* functions have been identified in some species. For example, it was shown that *takeout* (named *Deviate*) elicited trail-following behavior in *R. flavipes*^19^. In *R. aculabialis*, *takeout* (named *RaSsp1*) was highly expressed in soldiers and was suggested to interact with JH III and participate in soldier differentiation^20^. In the termitid termite *Nasutitermes takasagoensis*, *takeout* (named *Ntsp1*) is highly expressed in the soldier frontal gland and probably combines with gland secretions^21^. Moreover, several *takeout* genes (named *takeout* -*3*, -*4* and -*9*) are highly expressed in the queens of *Cryptotermes secundus*, which might reflect the high JH titer levels^22^. However, in any species of termites, expression and function of *takeout* paralogs are not clearly understood.

Consequently, to provide additional information for understanding the importance of gene duplication during termite social evolution, we focused on *takeout* genes in *R. speratus*. First, based on genome and transcriptome data^9^, we searched *takeout* paralogs of *R. speratus* and inferred molecular phylogeny with homologs identified in termites and cockroaches. Second, we identified the full cDNA sequences of some paralogs and confirmed the presence of tandemly aligned paralogs within the same scaffold. Third, we performed real-time qPCR to determine the expression patterns of each *takeout* paralog among the three castes (reproductives, soldiers, and workers), two body parts (heads and remaining body parts), and sexes. Finally, we performed in situ hybridization to detect the expression sites of tandemly aligned paralogs with caste-specific expression patterns. Based on these results, we discuss the functionalization of *takeout* genes identified in the *R. speratus* genome.

## Materials and methods

### Identification of *takeout* genes

Blastp was performed to search for candidate *takeout* genes in the genome of *R. speratus* (RspeOGS1.0;^9^) using protein sequences of *D. melanogaster* JHBP genes (16 genes;^23^) as queries with an e-value cutoff of 1.0E − 05. We also ran a Hidden Markov Model (HMM) search using the HMM profile 'JHBP' (pfam06585) as a query. The obtained candidate genes were analyzed using InterProScan (https://www.ebi.ac.uk/interpro/;^24^) to confirm the conserved domains. Signal peptide regions were predicted using SignalP version 5.0 (https://services.healthtech.dtu.dk/service.php?SignalP-5.0;^25^). A heatmap was constructed using the heatmap.2 function included in the R package g plots (https://github.com/talgalili/gplots). Gene expression levels were compared among castes and between sexes using read per kilobase million (RPKM) calculated in a previous study^9^.

### Molecular phylogenetic analysis

To clarify the relationships between the two newly identified *takeout* genes (*RsTO1* and *RsTO2*; see below), we obtained homologous sequences from three blattodeans with genome information (termites: *Zootermopsis nevadensis* and *Cryptotermes secundus*; cockroaches: *Blattella germanica*) using protein sequences of *RsTO1* and *RsTO2* as queries. The top-hit *RsTO1* and *RsTO2* homologous sequences were obtained for each termite species, but the same sequence was selected in *B. germanica*. The remaining *takeout* genes obtained in *R. speratus* (total 22; see below) and those previously published in other termites (total six; described in the Introduction) were included in the analysis. Amino acid sequences were aligned using MAFFT^26^. Ambiguously aligned regions were removed from the analysis using trimAl with the “gappyout” option^27^ (Supplementary data S1). RAxML-NG^28^ was used for tree construction using the maximum likelihood method (bootstrap value 100). Optimal substitution models were estimated using ModelTest-NG^29^. The LG + G4 model was selected.

### Termites

Four mature colonies of *R. speratus* were collected from Toyama Prefecture, Japan, in 2019 and 2022. Colonies were maintained in plastic boxes and kept at 25 °C in constant darkness. One colony collected in 2019 was used as the source colony for the presoldiers. According to previous studies^30,31^, presoldiers and workers were maintained on moist, red-colored, No. 14 paper (Goukaseishi Co., Ltd., Aichi, Japan). Non-gut-purged individuals (with red-colored abdomens) were sampled. These presoldiers and workers were maintained in a petri dish containing moist filter paper. All dishes were incubated at 25 °C in constant darkness and checked every 24 h. Non-gut-purged and gut-purged presoldiers and soldiers 0, 2, 4, and 6 days after the molt were sampled from the dishes (total of three individuals per day). Whole-body specimens were immediately frozen in liquid nitrogen and stored at − 80 °C until use. Filter papers (55 mm diameter; Advantec, Tokyo, Japan) were treated with 160 µg JH III (Santa Cruz Biotechnology, Dallas, TX, USA) dissolved in 200 µL of acetone. According to a previous study^30^, non-gut-purged workers were exposed to filter paper in a petri dish. Gut-purged (soldier-destined) workers and induced molted presoldiers were obtained from the dishes, and natural soldiers were obtained from the colony and then fixed in formaldehyde:ethanol:acetic acid (6:16:1) solution for at least 24 h and preserved in 70% ethanol.

Three colonies collected in 2022 were used for RNA extraction from each caste (reproductive, soldier, and worker castes) and female individuals (last instar nymphs and alates). Nymphs were collected at two time points (several months before the swarming season, and just before the imaginal molt) from one colony. The latter nymphs had developed wing buds on the meso- and metanotum. Then the female alates were collected from the same colony within 7 days after the emergence. Primary reproductives (queens and kings) were collected from an incipient colony approximately 3 months (used for the expression analysis among castes) or 6 months (used for the expression analysis among females) after the colony foundation. Incipient colonies were constructed as previously described^32^. Sexes were identified using the morphological characteristics of the seventh and eighth abdominal sternites (workers and soldiers) or abdominal tergites (reproductives)^33–35^. Each individual was divided into head and body parts (including thorax and abdomen with guts) on ice. For the gene expression analysis among females, each individual was divided into head + thorax and abdomen with guts. Note that the abdomen of the reproductives normally contain the reproductive tissues (ovary and testis). Each tissue was immediately frozen in liquid nitrogen, and stored at − 80 °C until use.

### RNA extraction

RNA was extracted from each caste (reproductives, soldiers, and workers) using the ReliaPrep RNA Tissue Miniprep System (Promega, Madison, WI, USA). Total RNA was extracted from three individuals (heads or remaining body parts) per sample from three different colonies, and triplicate biological samples were prepared for each category (i.e., three individuals per sample, and one sample per colony × three colonies). RNA extraction from presoldiers and soldiers (whole-body) and female individuals (head + thorax, or abdomen) was performed using ISOGEN II (Nippon Gene, Tokyo, Japan). In this case, total RNA was extracted from three (presoldiers and soldiers) or two individuals (females) per sample from three different colonies (queens) or the same colony (presoldiers and soldiers were derived from one colony collected in 2019, and nymphs and alates were derived from another colony collected in 2022), and triplicate biological samples were prepared for each category [i.e., two individuals per sample, and one sample per colony × three colonies (queens); 2–3 individuals per sample, and three samples per colony × one colony (other developmental stages)]. Extracted RNA was purified using DNase I (TaKaRa Bio, Shiga, Japan). The quality and quantity of the purified RNA were measured using a NanoVue spectrophotometer (GE Healthcare Bio-Sciences, Tokyo, Japan) and a Qubit 2 fluorometer (Thermo Fisher Scientific, Waltham, MA, USA).

### Rapid amplification of cDNA ends (RACE)

Total RNA was extracted from five soldiers and five workers using ISOGEN II (Nippon Gene). The extracted RNA was purified by DNase treatment, using the method described above. According to a previous study^12^, cDNA was synthesized using the SMARTer RACE cDNA amplification kit (Clontech Laboratories, Mountain View, CA, USA). Gene-specific primers were designed from cDNA sequences (gene ID: RS000936) using Primer3Plus^36^ (Supplementary Table S1). The obtained 5ʹ- and 3ʹ-RACE products were purified using a QIAquick Gel Extraction Kit (QIAGEN, Tokyo, Japan), subcloned into a pGEM easy T-vector (Promega), and transfected into *Escherichia coli* XL1-blue. Plasmids with the target DNA fragments were extracted from a single colony of *E. coli* using the SIGMA GelEluteTM Plasmid Miniprep Kit (Sigma-Aldrich, St. Louis, MO, USA). The inserted DNA sequence was determined using the BigDye Terminator v3.1 Cycle Sequencing Kit (Thermo Fisher Scientific) or QuantumDye Terminator Cycle sequencing Kit v3.1 (Tomy Digital Biology Co., Ltd., Tokyo, Japan) with an automatic DNA Sequencer 3130 Genetic Analyzer or Applied Biosystems 3500 Serises Genetic Analyzer (Thermo Fisher Scientific). The obtained cDNA sequence was confirmed using a BLAST search and deposited in the DDBJ/EMBL/GenBank databases (accession no. LC742508 for *RsTO1* and LC742509 for *RsTO2*).

### Real-time qPCR

cDNA was synthesized from purified RNA using a High-Capacity cDNA Reverse Transcription Kit (Thermo Fisher Scientific). Gene expression levels of 11 genes (*RsTO1*, *RsTO2*, *RsVg1*, *RsVg2*, *GGPPS*, and six control genes; see below) were measured using Thunderbird SYBR qPCR Mix (Toyobo, Osaka, Japan) and the Mx3005P Real-Time QPCR System (Agilent Technologies, San Diego, CA, USA) (presoldiers/soldiers) or QuantStudio 3 Real-Time PCR System (Thermo Fisher Scientific) (castes and female individuals). To determine an internal control gene according to previous studies^12,37^, the suitability of six reference genes was evaluated using GeNorm^38^ and NormFinder^39^ software: *EF1-alfa* (accession no. AB602838), *NADH-dh* (no. AB602837), *β-actin* (no. AB520714), *GstD1* (gene ID: RS001168), *EIF-1* (RS005199), and *RPS18* (RS015150). Gene-specific primers were designed using Primer3Plus^36^ (Supplementary Table S1).

### In situ hybridization

Gene-specific primers were designed against the obtained *RsTO1* and *RsTO2* sequences using Primer3Plus^36^ for RNA probe synthesis (Supplementary Table S1). Total RNA was extracted from heads + thoraxes of two queens and two female alates (for *RsTO1* probe) and whole bodies of five soldiers (for *RsTO2* probe) using ISOGEN II (Nippon Gene) and purified as described above. cDNA was synthesized as described above and used to amplify the *RsTO1* and *RsTO2* fragment. The amplified *RsTO1* and *RsTO2* fragments were subcloned and the inserted DNA sequences were determined using the sequencing method described above. The digoxygenin (DIG)-labeled RNA probes for *RsTO1* and *RsTO2* were prepared from a plasmid containing each fragment using a DIG RNA Labeling Kit (Roche, Basel, Switzerland). Two alates and three soldiers were separated into their body parts by dissection, and each part (alates: head + thorax; soldiers: head) was fixed with 4% paraformaldehyde and cryoprotected in sucrose solution. The fixed samples were embedded in OCT compound (Sakura Finetek USA Inc., Torrance, CA, USA) and sliced to prepare 9 µm (alates) or 10 μm thick (soldiers) parasagittal cryosections using a CM1510S cryostat (Leica, Tokyo, Japan). The sections were hybridized with DIG-labeled sense or antisense RNA probes using In Situ Hybridization Reagents (Nippon Gene), in accordance with the manufacturer’s instructions. DIG-labeled RNA was detected using a DIG Nucleic Acid Detection Kit (Roche). Images were captured using a Biozero microscope (Keyence, Tokyo, Japan).

### Histological observations

Paraffin-embedded sections of female alates were used to observe the frontal glands, normally restricted to the heads^40,41^. Those of heads of induced presoldiers and natural soldiers were used to observe the frontal glands. Samples preserved in 70% ethanol were dehydrated in increasing concentrations of ethanol, transferred into xylene, and embedded in paraffin. Serial parasagittal sections (6 μm thick) were processed using an MRS80-074 microtome (Ikemoto, Tokyo, Japan) and stained with hematoxylin and eosin. Tissues on the slides were observed under a Biozero microscope (Keyence).

### Statistical analysis

Expression levels of *RsTO1* and *RsTO2* (shown below) were compared among castes (reproductives, soldiers and workers) and sexes (females and males) using two-way ANOVA followed by post hoc multiple comparison test (Tukey’s test, *p* < 0.05). Those of *RsTO1*, *RsTO2*, *RsVg1*, *RsVg2*, and *GGPPS* (shown below) were compared among developmental stages (*RsTO1*, *RsVg1*, and *RsVg2*: last-instar nymphs, alates and primary reproductives; *RsTO2* and *GGPPS*: presoldiers and soldiers) using one-way ANOVA followed by post hoc multiple comparison test (Tukey’s test, *p* < 0.05). Statistical analyses were performed separately for each part of the body. We performed Levene’s test or Bartlett test on variance equality and confirmed that the data was basically consistent with the use of parametric statistics. All of these tests were performed using Mac Statistical Analysis ver. 3.0 (Esumi, Tokyo, Japan).

## Results

### *Takeout* genes identified in *R. speratus*

Thirty-two *takeout* genes were identified in the *R. speratus* genome (Rspe OGS1.0;^9^ (Supplementary Table S2). We confirmed that all genes contained 'Hemolymph juvenile hormone binding' (IPR010562) and 'Takeout superfamily' (IPR038606) using the InterProScan database. However, there were five genes with very short sequences (< 250 bases, only one exon) and two genes with large distances between exons (> 20,000 bases). In the latter case (*RS000936*), to clarify the possibility of insufficient gene annotation, we performed several 5'- and 3'-RACE analyses using gene-specific primers (Supplementary Table S1). We then confirmed that there were two genes in the focal regions of scaffold_1087 (named *RsTO1* and *RsTO2*, respectively) (Supplementary Figure S1). We could not find a secretion signal peptide in the N-terminal region in a total of three genes with an initial methionine (Supplementary Table S2). We confirmed that both *RsTO1* and *RsTO2* contained a Takeout superfamily motif and a secretion signal peptide (likelihood of signal peptide: 0.5951 and 0.9975, respectively; Supplementary Table S2). In sum, 25 genes (= 32 minus 7) were used for the comparative analysis of expression levels using RNA-seq data, and 27 genes (= 25 plus *RsTO1* and *RsTO2*) were used for the phylogenetic analysis.

### Molecular phylogeny of *R. speratus takeout* genes

Molecular phylogeny analysis revealed that *RsTO1* and *RsTO2* were not closely related (Supplementary Figure S2). Termites (at least two species with genomic information) had homologs of both *RsTO1* and *RsTO2*, and orthologous relationships were observed. *Takeout* genes previously published in the genus *Reticulitermes* (i.e., *R. aculabialis RaSsp1* and *R. flavipes Deviate*) were most closely related to *RsTO2* and *RS013761*, respectively (bootstrap value 100%). *Takeout* genes reported in *N. takasagoensis* (named *Ntsp1*) showed a sister group relationship with the clade containing *RsTO2*. *Takeout* genes reported in *C. secundus* (*takeout-3* and -*4*) were most closely related to *RS014379* and *RS013482*, respectively (bootstrap values 98–100%). A remaining *takeout* in *C. secundus* (*takeout-9*) was closely related to *RS002171*, but the support of this branch was not strong (bootstrap values below 50%).

### Expression patterns of *takeout* genes among castes

**Figure 1:**
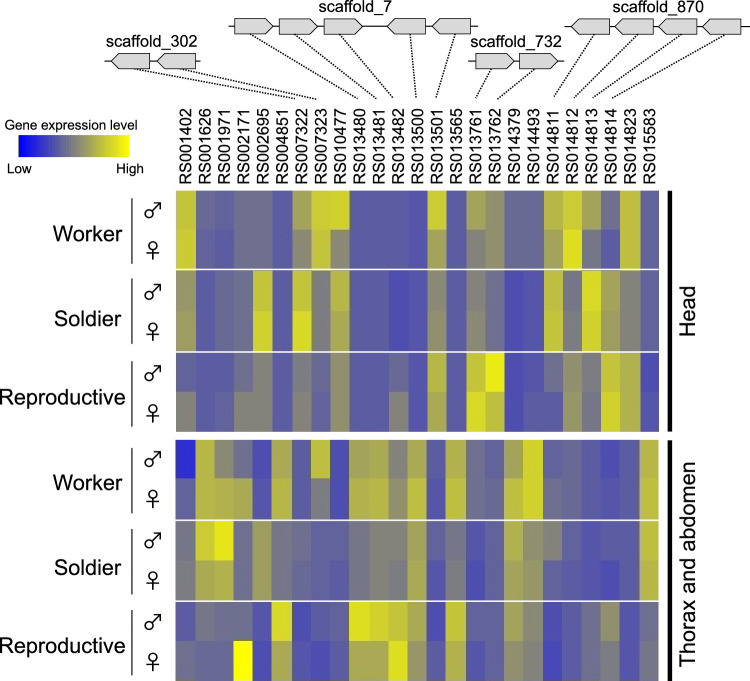
Genomic location and gene expression of *R. speratus takeout* genes. Heatmap of Z-scores of the log (RPKM + 1) values of *takeout* genes (total 25) in the caste-specific transcriptome^9^. Yellow and blue indicate high and low expression, respectively. Black represents the mean level of expression among castes.

**Figure 2:**
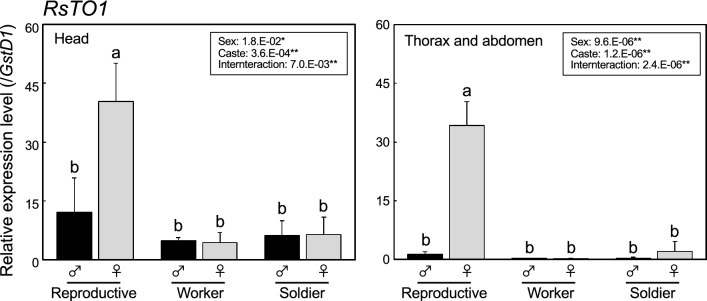
Quantitative real-time PCR expression levels (mean ± S.D., biological triplicates) of *RsTO1* in head (left) and thorax and abdomen (right) among primary reproductives, workers and soldiers. Total RNA was extracted from three individuals per sample in each colony, and three biological samples (derived from different colonies) were prepared. Each value is normalized to the expression levels of *GstD1* (Supplementary Table S3). Black and grey columns indicate males and females, respectively. Statistical results of the two-way ANOVA are described in each box (*P < 0.05, **P < 0.01). Different letters above the bars indicate significant differences (two-way ANOVA followed by Tukey’s test, *P* < 0.05).

**Figure 3:**
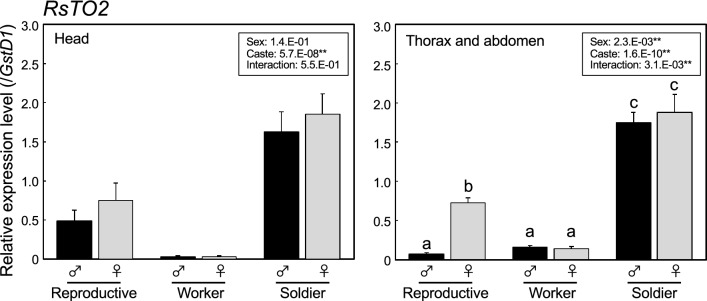
Quantitative real-time PCR expression levels (mean ± S.D., biological triplicates) of *RsTO2* in head (left) and thorax and abdomen (right) among primary reproductives, workers and soldiers. Total RNA was extracted from three individuals per sample in each colony, and three biological samples (derived from different colonies) were prepared. Each value is normalized to the expression levels of *GstD1* (Supplementary Table S3). Black and grey columns indicate males and females, respectively. Statistical results of the two-way ANOVA are described in each box (*P < 0.05, **P < 0.01). Different letters above the bars indicate significant differences (Two-way ANOVA followed by Tukey’s test, *P* < 0.05). We could not perform Tukey's test in the left graph, because interaction was not detected between caste and sex (*p* = 5.5.E − 01).

Using RNA-seq data^9^, the expression patterns of 25 genes were compared among the castes (Fig. 1, Supplementary Figure S3). The gene expression levels of *RsTO1* and *RsTO2* were analyzed by real-time qPCR (Fig. 2 and 3). *GstD1* was selected as the internal control gene because of its stability among the six genes analyzed (Supplementary Table S3). The expression patterns were significantly different for each gene. For example, there were some genes with high expression levels in soldiers (e.g., *RsTO2* and *RS007322*), but those with low expression levels in soldiers (e.g., *RS013480* and *RS013481*). Moreover, there were some genes with high expression levels in workers (e.g., *RS007323*) and reproductives (e.g., *RsTO1* and *RS013482*). If the gene duplication is involved in the social evolution^9^, caste-specific expression patterns should be observed even among tandem-aligned and recent duplicated paralogs. To clarify this possibility, tandem-aligned genes, such as *RS013480*-*2* (scaffold_7, Fig. 1) and *RsTO1* and *RsTO2* (scaffold_1087; Supplementary Figure S1), should be focused.

### Expression patterns of *RsTO1* and *RsVgs* among female individuals

**Figure 4:**
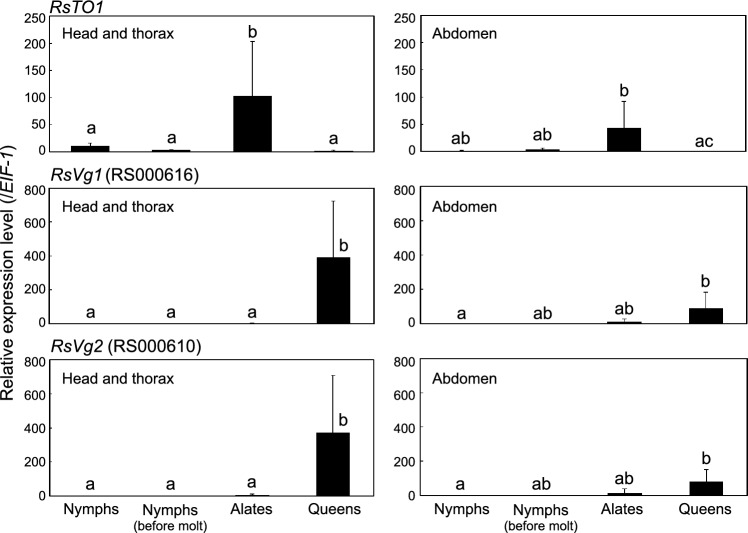
Quantitative real-time PCR expression levels (mean ± S.D., biological triplicates) of *RsTO1* (upper), *RsVg1* (middle), and *RsVg2* (lower) in head and thorax (left) and abdomen (right) among female last-instar nymphs (several months before and just before the imaginal molt), alates (within 7 days after the imaginal molt), and queens (approximately 6 months after colony foundation). Total RNA was extracted from two individuals per sample in each colony, and three biological samples (derived from different colonies (queens) or the same colony (nymphs and alates)) were prepared. Each value is normalized to the expression level of *EIF-1* (Supplementary Table S4). Different letters above the bars indicate significant differences (One-way ANOVA followed by Tukey’s test, *P* < 0.05).

We attempted to clarify the expression patterns of *RsTO1* among female individuals; last instar nymphs (several months before the imaginal molt, just before the imaginal molt), alates (within 7 days after the emergence), and queens (about 6 months after the colony foundation). We also analyzed the expression patterns of *Vitellogenin* (*RsVg1*, gene ID: RS000616, *RsVg2*, gene ID: RS000610), both of which are highly expressed in fertile queens and mRNA of the former is observed in the fat body of a queen^12^. *EIF-1* was selected as the internal control gene because of its stability among the six genes analyzed (Supplementary Table S4). The expression levels of all three genes examined were low in the last instar nymphs (both nymphs collected several months and just before the imaginal molt; Fig. 4). As shown in the previous study, the expression levels of both *RsVg1* and *RsVg2* were higher in queens. In contrast, the expression levels of *RsTO1* were significantly higher in alates but extremely low in queens. Similar expression patterns were observed in different tissues (head + thorax, abdomen) in all three genes.

### Expression patterns of *RsTO2* and *GGPPS* during soldier formation

**Figure 5:**
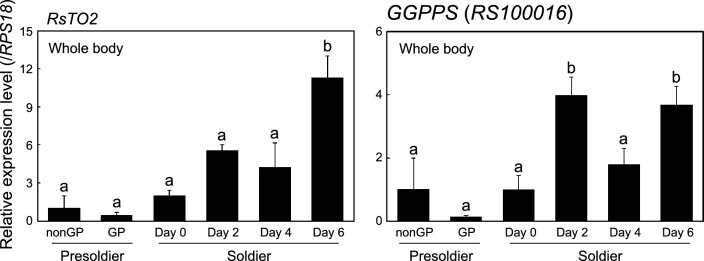
Quantitative real-time PCR expression levels (mean ± S.D., biological triplicates) of *RsTO2* (left) and *GGPPS* (right) during presoldier and soldier differentiation (whole-body). Total RNA was extracted from three individuals per sample, and three biological samples (derived from the same colony) were prepared. Each value is normalized to the expression level of *RPS18* (Supplementary Table S5). GP means gut purge. Different letters above the bars indicate significant differences (One-way ANOVA followed by Tukey’s test, *P* < 0.05).

We attempted to clarify the expression patterns of *RsTO2* during presoldier and soldier formation. We also analyzed the expression patterns of *geranylgeranyl pyrophosphate synthase* (*GGPPS*, gene ID: RS100016), which is highly expressed in the soldier frontal glands^9^. Soldier-specific expressed *GGPPS* might be involved in the synthesis of glandular defensive substances, such as diterpenes^42,43^. *RPS18* was selected as the internal control gene because of its stability among the six genes analyzed (Supplementary Table S5). The expression levels of both *RsTO2* and *GGPPS* were relatively low in presoldiers (Fig. 5). However, after soldier molt, the expression levels of both genes tended to be higher. *RsTO2* in soldiers 6 days after molting exhibited significantly higher expression levels than any other stage. Similar expression patterns were observed in *GGPPS*, and the highest expression levels were obtained in soldiers after molting (days 2 and 6).

### In situ hybridization of *RsTO1*

**Figure 6:**
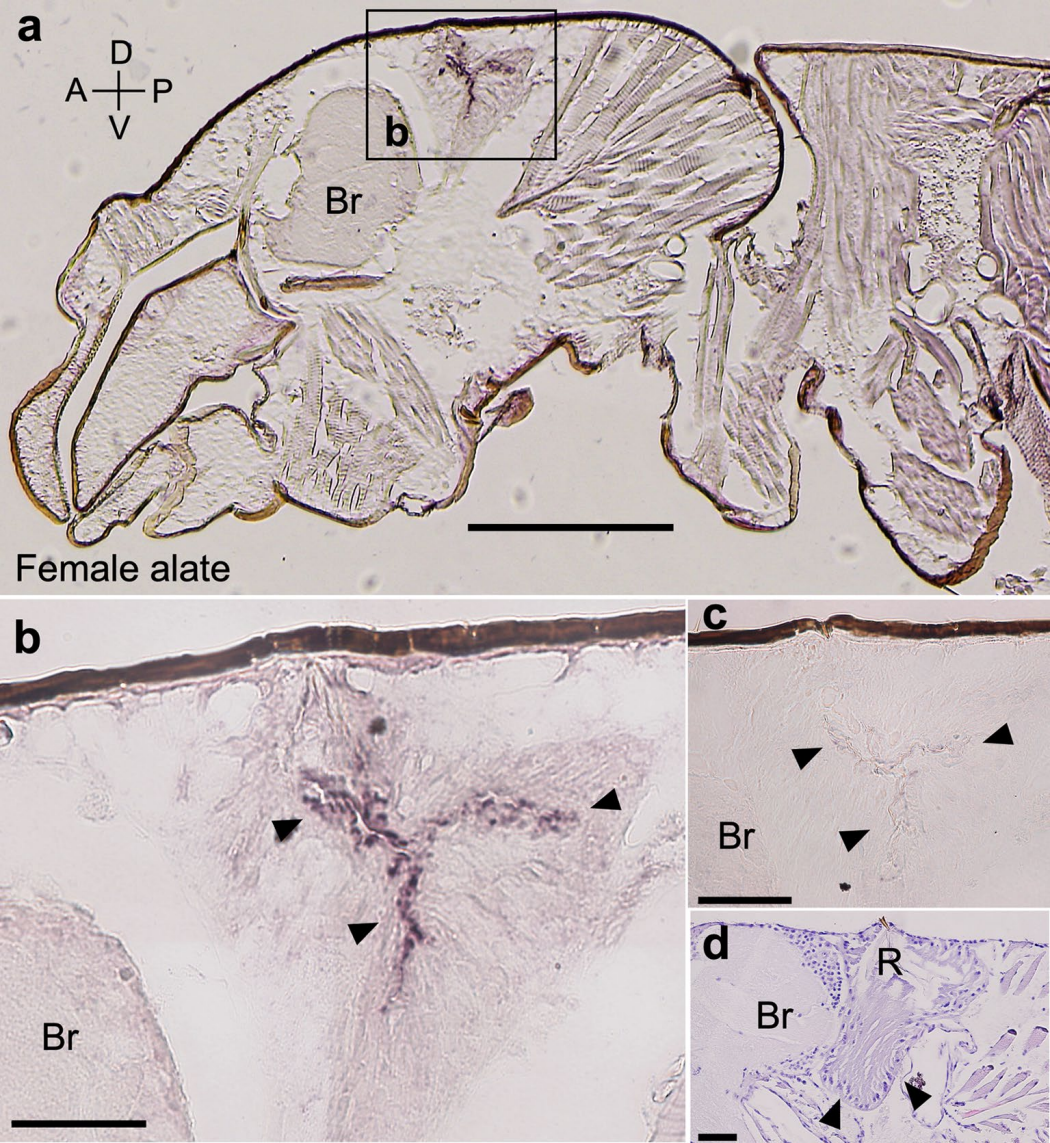
In situ hybridization of *RsTO1* mRNA in the alate (female) head and thorax of *R. speratus*. Cryosections (9-µm thick) of an alate head and thorax were subjected to in situ hybridization with an antisense DIG-labeled RNA probe (**a** and **b**). Glandular cells hybridized with a sense probe (negative control) (**c**). Arrowheads indicate the gland cell layers. Sagittal sections (6-µm thick) of the frontal glands of female alates (**d**, arrowhead). Orientations of all slides are indicated in (**a**). A: Anterior, P: Posterior, D: Dorsal, V: Ventral. Br: Brain, R: Reservoir of the frontal gland. Bars = 0.3 mm (**a**) and 0.05 mm (**b**, **c**, and **d**).

*RsTO1* mRNA localization was examined by in situ hybridization of heads and thoraxes of female alates (Fig. 6a,b,c). *RsTO1* mRNA signals were confirmed to localize in the frontal glands located in the dorsal region of the heads (Fig. 6a,b). Specific signals were not observed in sections treated with the sense probe (Fig. 6c). Histological observations using paraffin-embedded sections indicated that a saculiform reserver was surrounded by the glandular epithelium made up of frontal gland cells (Fig. 6d).

### In situ hybridization of *RsTO2*

**Figure 7:**
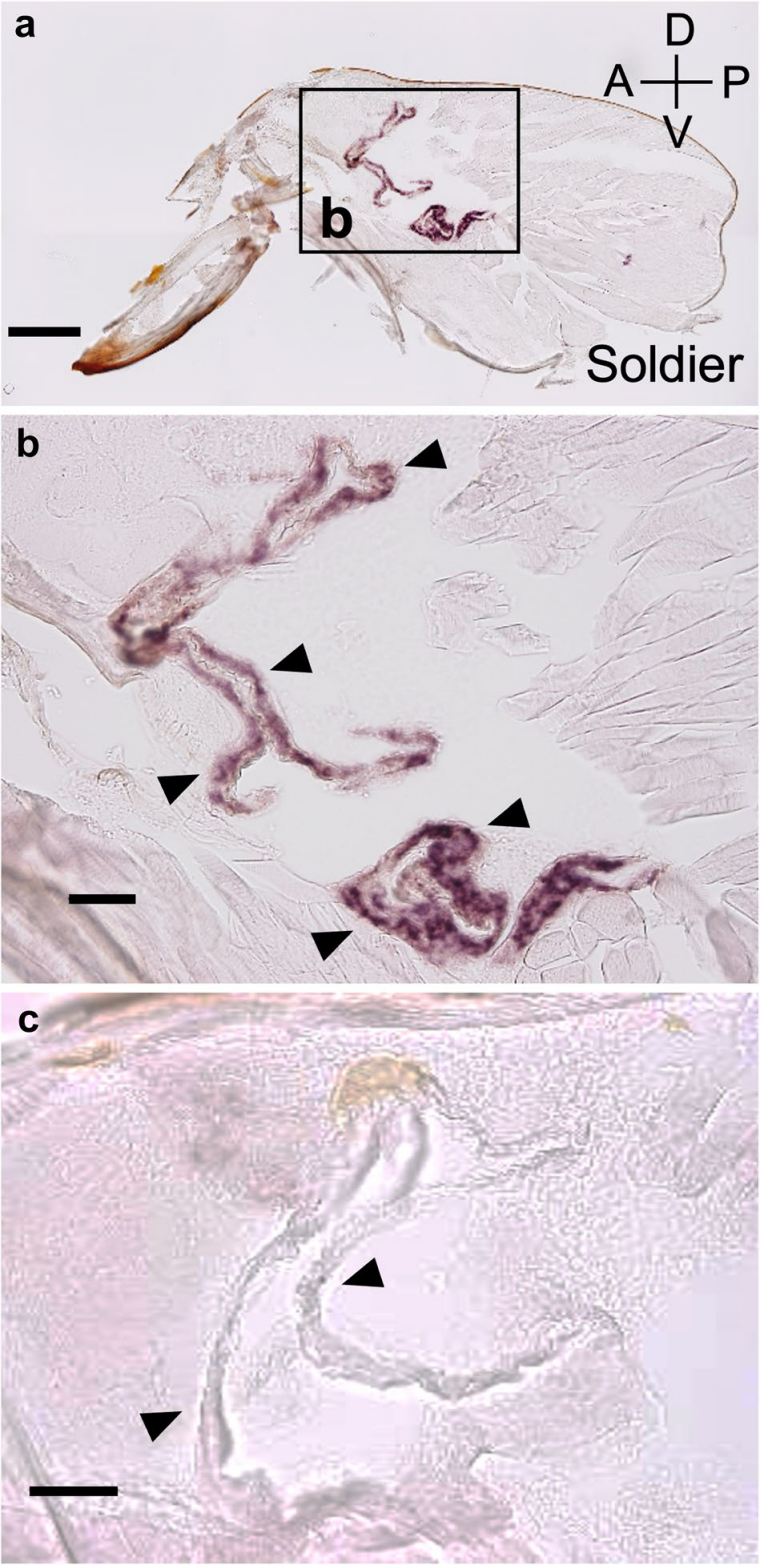
In situ hybridization of *RsTO2* mRNA in the soldier head of *R. speratus*. Cryosections (10-µm thick) of a soldier head were subjected to in situ hybridization with an antisense DIG-labeled RNA probe (**a** and **b**). Glandular cells hybridized with a sense probe (negative control) (**c**). Arrowheads indicate the gland cell layers. Orientations of all slides are indicated in (**a**). A: Anterior, P: Posterior, D: Dorsal, V: Ventral. Bars = 0.2 mm (**a**) and 0.05 mm (**b** and **c**).

*RsTO2* mRNA localization was examined by in situ hybridization of soldier heads (Fig. 7a,b). *RsTO2* mRNA signals were localized in the frontal glands of soldiers. Specific signals were not observed in sections treated with the sense probe (Fig. 7c).

### Observation of frontal glands of soldiers

**Figure 8:**
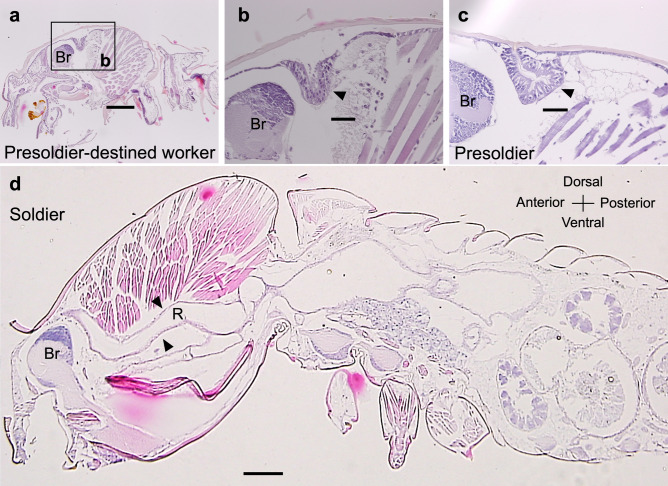
Sagittal sections (6-µm thick) of the worker, presoldier and soldier heads of *R. speratus*. Frontal glands (arrowhead) of a presoldier-destined worker (gut-purged worker after JH treatment) (**a** and **b**), a presoldier induced by JH treatment (**c**), and a natural soldier (**d**). Orientations of all slides are indicated in (**d**). Br: Brain, R: Reservoir of the frontal gland. Bars = 0.2 mm (**a** and **d**), 0.05 mm (**b** and **c**).

To clarify the degree of invagination and cell proliferation in frontal glands of soldier-destined workers and presoldiers, we performed the histological observations using paraffin-embedded sections of heads. Frontal glands were observed using parasagittal sections of gut-purged (soldier-destined) workers, induced presoldiers, and natural soldiers (Fig. 8). Frontal gland cells invaginated from the frontal pores were already initiated in the head of gut-purged (soldier-destined) workers (Fig. 8a,b). As shown in previous studies^43^, the frontal gland cavity was constructed in presoldiers, and frontal gland reservoir was finally observed in soldiers (Fig. 8c,d).

## Discussion

### Expression of *takeout* genes in *R. speratus*

We confirmed that all 32 genes obtained from *R. speratus* genome (OGS1.0;^9^) contained the JHBP domain. However, sequences that were too short (< 250 bases) and genes with extraordinarily long distances between exons (> 20,000 bases) were present (Supplementary Table S2). It is possible that the predictions of such genes are not sufficient. Indeed, of the newly identified *RsTO1* and *RsTO2*, the former contained most *RS000936* and the latter the 3' region of *RS000936*. Comparison of the final genome assembly^9^ and gene sequences obtained by RACE revealed at least two regions with many N’ (i.e., A, T, G, or C) in the genome assembly (Supplementary Figure S1). This may explain the inaccurate prediction of *RS000936*. Further analysis was performed to obtain the complete cDNA sequences of the other six genes listed in Supplementary Table S2. We suggest that there were no pseudogenes in 25 genes remaining, because all of which showed evidence of expression with a threshold of RPKM = 1.0 in at least one sample of RNA-seq data (Supplementary Figure S3).

The expression patterns of all *takeout* genes (25 genes explained above, and *RsTO1* and *RsTO2*) differed among castes (Figs. 1, 2, 3). There were at least five examples of multiple genes in the same scaffold (Fig. 1, Supplementary Figure S1). However, molecular phylogeny showed that these genes on the same scaffold were not closely related (Supplementary Figure S2). It is possible that these are additional examples of functionalization caused by gene duplication^9^. We then focused on the expression of *RsTO1* and *RsTO2*. Their roles are discussed next. Molecular phylogeny analysis also indicated that each termite species had orthologs for both *RsTO1* and *RsTO2* (Supplementary Figure S2). However, in the German cockroach *B. germanica*, only one homolog was obtained within the clade containing *RsTO1*, although branch support was very weak (53% bootstrap values). To clarify the possibility that gene duplication events occurred in the termite lineage after splitting from the common ancestor of cockroaches, homologous sequences of cockroaches should be searched for, especially in the sister group of termites, the woodroach *Cryptocercus* spp.

### Prediction of functions of *RsTO1*

*RsTO1* was highly expressed in queens, compared to other castes (both head and body) (Fig. 2). Homologous genes were found in *C. secundus* and *Z. nevadensis* and the monophyly of these three genes was strongly supported (97% bootstrap values; Supplementary Figure S2), suggesting that these genes have a common function. One possibility for this function is that RsTO1 is involved in high JH titers in termite queens, which has been confirmed in some species, including *R. speratus*^32,44^. We also confirmed that *RsVg1* and *RsVg2*, both of which may have JH-related expression patterns and work for female vitellogenesis^12^, were highly expressed in queens (Fig. 4). In *C. secundus*, three *takeout* genes (named *takeout* -*3*, -*4* and -*9*) were included in the queen-specific expressed genes (Queen Central Module; QCM) and were suggested to be involved in JH transport, reproduction, and longevity in queens^22^. Molecular phylogenetic analysis showed that *RsTO1* was not closely related to any of these three genes in *C. secundus*. However, orthologs of *takeout-3* and -*4* were found in *R. speratus* (*RS014379* and *RS013482*, respectively). To clarify whether there are species-specific and diverse roles for *takeout* genes of QCM, we should investigate the gene expression sites of these homologs and compare the differences between species.

Alternatively, we suggest that *RsTO1* has specific roles in female alates. Expression patterns of *RsTO1* were considerably changed during queen differentiation, and expression levels were particularly high in alates (especially in head + thorax; Fig. 4). Expression patterns of *RsTO1* and *RsVgs* were different in queens, suggesting that *RsTO1* was not expressed in developed reproductive organs. Indeed, in situ hybridization showed that *RsTO1* mRNA was localized in the frontal glandular cells of female alates (Fig. 6). Chemical analysis has not been performed in *R. speratus*, but a complex mixture mainly derived from terpenes were detected from the frontal glands of alates in the rhinotermitid termite *Prorhinotermes* species^45^. Present results suggest that RsTO1 is involved in binding with these chemicals produced in the frontal glands of female alates. The most plausible function of secretions is the defense against the predation during swarming flights^45^. However, it is still unclear why the female-specific *RsTO1* expression patterns are observed in *R. speratus*. One possibility is that the calling and mating behavior of female alates is regulated by *RsTO1*, especially highly expressed in the abdomens of female alates (Fig. 4). Although, in many termites, the sexual attraction is regulated by the secretion from tergal glands of alates (e.g.,^46^), these glands could not be observed in *R. lucifugus*^47^. Consequently, to know the female-specific *RsTO1* functions in *R. speratus*, further in situ analysis should be performed in the abdomens of alates. Moreover, alates (and also soldiers) do not possess frontal glands in *Z. nevadensis* and *C. secundus*^48^. If *RsTO1* homologs, such as *Z. nevadensis* XP_021917347.1 and *C. secundus* XP_023709343.1 (Supplementary Figure S2), are also specifically expressed in alates of both species, gene expression sites and related functions should be changed. Further analyses are needed to clarify this possibility.

### Prediction of functions of *RsTO2*

In situ hybridization showed that *RsTO2* mRNA was localized in the frontal glandular cells of the soldiers (Fig. 7). Expression levels of *RsTO2* were low in presoldiers but were significantly increased 6 days after the presoldier-soldier molt (Fig. 5). Both the invagination of the frontal gland and the formation of glandular cells might not be completed in presoldiers (Fig. 8). Thus, *RsTO2* may exhibit gene expression and function after synthesis of defensive substances in the mature frontal gland in soldiers. The results of *GGPPS* expression levels, which preceded those of *RsTO2* (Fig. 5), support this possibility. In the nasute termite *N. takasagoensis*, the *takeout* gene (*Ntsp1*) is specifically expressed in the soldier frontal gland and was suggested to bind with some terpenoids (including diterpenes) for colony defensive substances and/or alarm pheromones^21^. The expression patterns and amino acid sequences of *Ntsp1* were very similar to those of *RsTO2*, suggesting that these genes have common functions. In *Reticulitermes* spp., some diterpenes are produced in the frontal gland^49,50^. Moreover, in *R. speratus*, terpenoids (β-elemene) have been identified in soldier extracts^51^. It is possible that RsTO2 binds to various terpenoids and is involved in the retention of their activity and inhibition of their decomposition and/or transport within the frontal gland cavity. Interestingly, *RS002695* and *RS001626* were observed in the same clade as *RsTO2*, and both genes tended to be highly expressed in soldiers (Fig. 1, Supplementary Figure S3). Expression analyses should be performed for these genes to clarify whether they have diverse *takeout* roles in soldiers. Moreover, as discussed in the previous section, soldiers do not possess frontal glands in *Z. nevadensis* and *C. secundus*^48^. Further analyses are needed to clarify whether gene expression sites and related functions of *RsTO2* homologs (XP_021914758.1 and XP_033607719.1; Supplementary Figure S2) are also changed in soldiers of *Z. nevadensis* and *C. secundus*.

The *takeout* gene reported in *R. aculabialis* (*RaSsp1*), which is most closely related to *RsTO2* (Supplementary Figure S2), may bind to JH III and is involved in the regulation of soldier differentiation^20^. To clarify whether RsTO2 interacts with JH III before molts and is involved in soldier differentiation, functional analysis should be performed during presoldier and soldier differentiation under natural conditions without hormone treatment.

## Conclusion

We obtained 27 *takeout* genes in *R. speratus*, including orthologs of those previously published in other termites (e.g., *RsTO2*: *RaSsp1* of *R. aculabialis*; *RS013761*: *Deviate* of *R. flavipes*; *RS014379* and *RS013482*: *takeout-3* and *-4* of *C. secundus*, respectively). Gene expression patterns among the castes were different for each gene, and some genes were tandemly aligned in the same scaffold. At least two paralogs were differentially expressed in frontal glands of female alates and soldiers. In termites, JH titer changes are crucial for caste differentiation^14^ and the glandular substances are important for their social system^41^. Thus, functionalization of *JHBP takeout* caused by gene duplication might be very important for social evolution in termites.

## Supplementary Information

Supplementary Information.

## Data Availability

DDBJ/EMBL/GenBank accession numbers for *takeout* genes newly obtained are LC742508 (*RSTO1*) and LC742509 (*RsTO2*).

## References

[1] Inward D, Beccaloni G, Eggleton P (2007). Death of an order: A comprehensive molecular phylogenetic study confirms that termites are eusocial cockroaches. Biol. Lett..

[2] Lo N (2000). Evidence from multiple gene seqeunces indicates that termites evolved from wood-feeding cockroaches. Curr. Biol..

[3] Roisin Y, Abe T, Bignell DE, Higashi M (2000). Diversity and evolution of caste patterns. Termites: Evolution, Sociality, Symbioses, Ecology.

[4] Korb J, Thorne B, Rubenstein DR (2017). Sociality in termites. Comparative Social Evolution.

[5] Miura T, Scharf ME, Bignell D, Roisin Y, Lo N (2011). Molecular basis underlying caste differentiation in termites. Biology of Termites: A Modern Synthesis.

[6] Hartfelder, K. & Emlen, D. J. Endocrine control of insect polyphenism. In: *Insect Endocrinology*, pp. 464–522. (Elsevier, 2012).

[7] Terrapon N (2014). Molecular traces of alternative social organization in a termite genome. Nat. Commun..

[8] Harrison MC (2018). Hemimetabolous genomes reveal molecular basis of termite eusociality. Nat. Ecol. Evol..

[9] Shigenobu S (2022). Genomic and transcriptomic analyses of the subterranean termite *Reticulitermes speratus*: Gene duplication facilitates social evolution. Proc. Natl. Acad. Sci..

[10] Maekawa K, Hayashi Y, Lo N (2022). Termite sociogenomics: Evolution and regulation of caste-specific expressed genes. Curr. Opin. Insect Sci..

[11] Wu T, Dhami GK, Thompson GJ (2018). Soldier-biased gene expression in a subterranean termite implies functional specialization of the defensive caste. Evol. Dev..

[12] Yaguchi H (2022). Evolution and functionalization of vitellogenin genes in the termite *Reticulitermes speratus*. J. Exp. Zool. B Mol. Dev. Evol..

[13] Peng X, Wang S, Huang L, Su S, Chen M (2021). Characterization of *Rhopalosiphum padi takeout-like* genes and their role in insecticide susceptibility. Pestic Biochem. Physiol..

[14] Korb J (2015). Juvenile hormone. A central regulator of termite caste polyphenism. Adv. Insect Physiol..

[15] Sarov-Blat L, So WV, Liu L, Rosbash M (2000). The *Drosophila takeout* gene is a novel molecular link between circadian rhythms and feeding behavior. Cell.

[16] Noriega FG (2006). Comparative genomics of insect juvenile hormone biosynthesis. Insect Biochem. Mol. Biol..

[17] Hagai T, Cohen M, Bloch G (2007). Genes encoding putative Takeout/juvenile hormone binding proteins in the honeybee (*Apis mellifera*) and modulation by age and juvenile hormone of the takeout-like gene *GB19811*. Insect Biochem. Mol. Biol..

[18] Du J, Hiruma K, Riddiford LM (2003). A novel gene in the *takeout* gene family is regulated by hormones and nutrients in *Manduca* larval epidermis. Insect Biochem. Mol. Biol..

[19] Schwinghammer MA, Zhou X, Kambhampati S, Bennett GW, Scharf ME (2011). A novel gene from the *takeout* family involved in termite trail-following behavior. Gene.

[20] Wu Z (2022). Soldier caste-specific protein 1 is involved in soldier differentiation in termite *Reticulitermes aculabialis*. Insects.

[21] Hojo M, Morioka M, Matsumoto T, Miura T (2005). Identification of soldier caste-specific protein in the frontal gland of nasute termite *Nasutitermes takasagoensis* (Isoptera: Termitidae). Insect Biochem. Mol. Biol..

[22] Lin S, Werle J, Korb J (2021). Transcriptomic analyses of the termite, *Cryptotermes secundus*, reveal a gene network underlying a long lifespan and high fecundity. Commun. Biol..

[23] Shin SW (2022). Inducible expression of several *Drosophila melanogaster* genes encoding Juvenile Hormone binding proteins by a plant diterpene secondary metabolite. Methyl Lucidone. Insects.

[24] Quevillon E (2005). InterProScan: Protein domains identifier. Nucleic Acids Res..

[25] Almagro Armenteros JJ (2019). SignalP 5.0 improves signal peptide predictions using deep neural networks. Nat. Biotechnol..

[26] Katoh K, Kuma KI, Toh H, Miyata T (2005). MAFFT version 5: Improvement in accuracy of multiple sequence alignment. Nucleic Acids Res..

[27] Capella-Gutiérrez S, Silla-Martínez JM, Gabaldón T (2009). trimAl: A tool for automated alignment trimming in large-scale phylogenetic analyses. Bioinformatics.

[28] Kozlov AM, Darriba D, Flouri T, Morel B, Stamatakis A (2019). RAxML-NG: A fast, scalable and user-friendly tool for maximum likelihood phylogenetic inference. Bioinformatics.

[29] Darriba D (2020). ModelTest-NG: A new and scalable tool for the selection of DNA and protein evolutionary models. Mol. Biol. Evol..

[30] Saiki R (2022). Comparison of gene expression profiles among caste differentiations in the termite *Reticulitermes speratus*. Sci. Rep..

[31] Masuoka Y, Miyazaki S, Saiki R, Tsuchida T, Maekawa K (2013). High *Laccase2* expression is likely involved in the formation of specific cuticular structures during soldier differentiation of the termite *Reticulitermes speratus*. Arthropod. Struct. Dev..

[32] Maekawa K, Ishitani K, Gotoh H, Cornette R, Miura T (2010). Juvenile hormone titre and vitellogenin gene expression related to ovarian development in primary reproductives compared with nymphs and nymphoid reproductives of the termite *Reticulitermes speratus*. Physiol. Entomol..

[33] Zimet M, Stuart AM (1982). Sexual dimorphism in the immature stages of the termite, *Reticulitermers flavipes* (Isoptera: Rhinotermitidae). Sociobiology.

[34] Hayashi Y, Kitade O, Kojima J (2003). Parthenogenetic reproduction in neotenics of the subterranean termite *Reticulitermes speratus* (Isoptera: Rhinotermitidae). Entomol. Sci..

[35] Weesner FM, Krishna K, Weesner FM (1969). External anatomy. Biology of Termites.

[36] Untergasser A (2007). Primer3Plus, an enhanced web interface to Primer3. Nucleic Acids Res.

[37] Miyazaki S (2021). Evolutionary transition of *doublesex* regulation from sex-specific splicing to male-specific transcription in termites. Sci. Rep..

[38] Vandesompele J (2002). Accurate normalization of real-time quantitative RT-PCR data by geometric averaging of multiple internal control genes. Genome Biol..

[39] Andersen CL, Jensen JL, Ørntoft TF (2004). Normalization of real-time quantitative reverse transcription-PCR data: A model-based variance estimation approach to identify genes suited for normalization, applied to bladder and colon cancer data sets. Cancer Res..

[40] Šobotník J, Bourguignon T, Hanus R, Sillam-Dusses D, Pflegerova J, Weyda F, Kutalová K, Vytisková B, Roisin Y (2010). Not only soldiers have weapons: Evolution of the frontal gland in imagoes of the termite families Rhinotermitidae and Serritermitidae. PLoS One.

[41] Costa-Leonardo AM, da Silva IB, Laranjo LT (2023). Termite exocrine systems: A review of current knowledge. Entomol. Exp. Appl..

[42] Hojo M, Shigenobu S, Maekawa K, Miura T, Tokuda G (2019). Duplication and soldier-specific expression of *geranylgeranyl diphosphate synthase* genes in a nasute termite *Nasutitermes takasagoensis*. Insect. Biochem. Mol. Biol..

[43] Hojo M, Toga K, Watanabe D, Yamamoto T, Maekawa K (2011). High-level expression of the *Geranylgeranyl diphosphate synthase* gene in the frontal gland of soldiers in *Reticulitermes speratus* (Isoptera: Rhinotermitidae). Arch. Insect. Biochem. Physiol..

[44] Saiki R, Gotoh H, Toga K, Miura T, Maekawa K (2015). High juvenile hormone titre and abdominal activation of JH signalling may induce reproduction of termite neotenics. Insect. Mol. Biol..

[45] Piskorski R (2009). Temporal and geographic variations in the morphology and chemical composition of the frontal gland in imagoes of *Prorhinotermes* species (Isoptera: Rhinotermitidae). Biol. J. Linn. Soc..

[46] Hanus R (2009). Sexual communication in the termite *Prorhinotermes simplex* (Isoptera, Rhinotermitidae) mediated by a pheromone from female tergal glands. Insectes Soc..

[47] Noirot C, Krishna K, Weesner FM (1969). Glands and secretions. Biology of Termites.

[48] Miura T, Maekawa K (2020). The making of the defensive caste: Physiology, development, and evolution of the soldier differentiation in termites. Evol. Dev..

[49] Quintana A (2003). Interspecific variation in terpenoid composition of defensive secretions of European *Reticulitermes* termites. J. Chem. Ecol..

[50] Nelson LJ, Cool LG, Forschler BT, Haverty MI (2001). Correspondence of soldier defense secretion mixtures with cuticular hydrocarbon phenotypes for chemotaxonomy of the termite genus *Reticulitermes* in North America. J. Chem. Ecol..

[51] Mitaka Y, Mori N, Matsuura K (2017). Multi-functional roles of a soldier-specific volatile as a worker arrestant, primer pheromone and an antimicrobial agent in a termite. Proc. R. Soc. B.

