# Supplementary material for: Caste-specific expressions and diverse roles of takeout genes in the termite Reticulitermes speratus
Source: Sci Rep. 2023 May 24;13:8422. doi: 10.1038/s41598-023-35524-7 (PMC10209217; doi:10.1038/s41598-023-35524-7)
Supplement: Supplementary file 1 — Supplementary Information. [file 41598_2023_35524_MOESM1_ESM.pdf]

Supplementary Information for:

Caste-specific expressions and diverse roles of *takeout* genes in the termite *Reticulitermes speratus*

Kokuto Fujiwara[1], Akimi Karasawa[2], Takumi Hanada[1], Mutsuaki Tobo[1], Tousuke Kaneko[2], Mizuna Usui[2] and Kiyoto Maekawa[3]

[1] Graduate School of Science and Engineering, University of Toyama, Gofuku, Toyama, 930-8555, Japan

[2] Department of Biology, Faculty of Science, University of Toyama, Gofuku, Toyama, 930-8555, Japan

[3] Faculty of Science, Academic Assembly, University of Toyama, Gofuku, Toyama, 930-8555, Japan

Table of Contents:

Figure S1. Original genome assembly of *RS000936* in the scaffold_1087, and possible exon/intron structures of *RsTO1* and *RSTO2*.	Page 001
Figure S2. Maximum likelihood (ML) tree of *takeout* homologs based on the amino acid sequences obtained with the LG+G4 model.	Page 002
Figure S3. Expression levels of 25 *takeout* genes among royals (reproductives), soldiers and workers in *R. speratus*.	Page 004
Table S1. Primer sequences used in this study.	Page 006
Table S2. Takeout genes identified in *Reticulitermes speratus*.	Page 007
Table S3. Stability values of reference genes in qPCR analysis among castes.	Page 008
Table S4. Stability values of reference genes in qPCR analysis among female nymphs, alates, and queens.	Page 009
Table S5. Stability values of reference genes in qPCR analysis during soldier formation.	Page 010
Data S1. Alignment of amino acid sequences of *takeout* genes used in this study.	Page 011

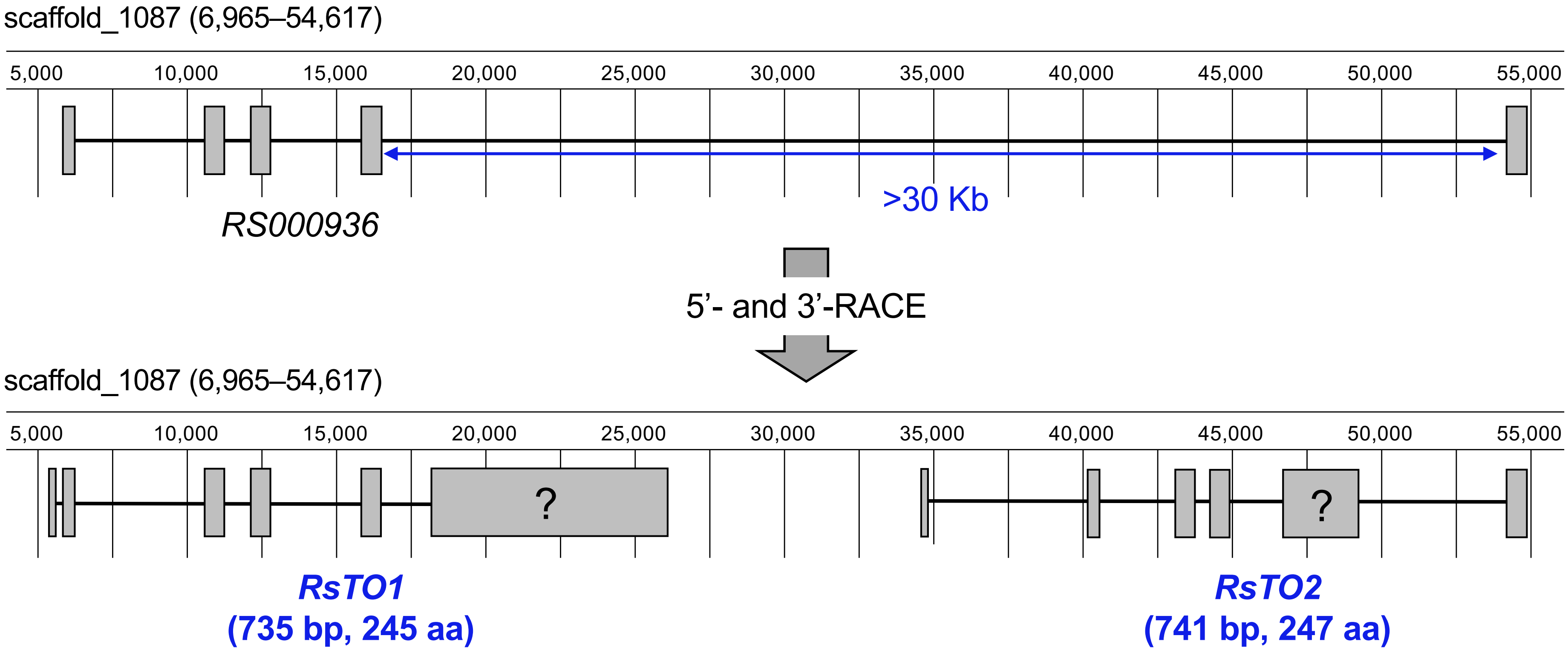

Fig. S1

Original genome assembly of *RS000936* in the scaffold_1087, and possible exon/intron structures of *RsTO1* and *RSTO2*. Two question marks indicate regions with ambiguous exon/intron structures due to the many N' (unknown DNA sequences) in the original scaffold_1087. The complete cDNA sequences of both *RsTO1* and *RSTO2* were obtained by RACE analysis. DDBJ accession numbers are listed in Supplementary Table S2.

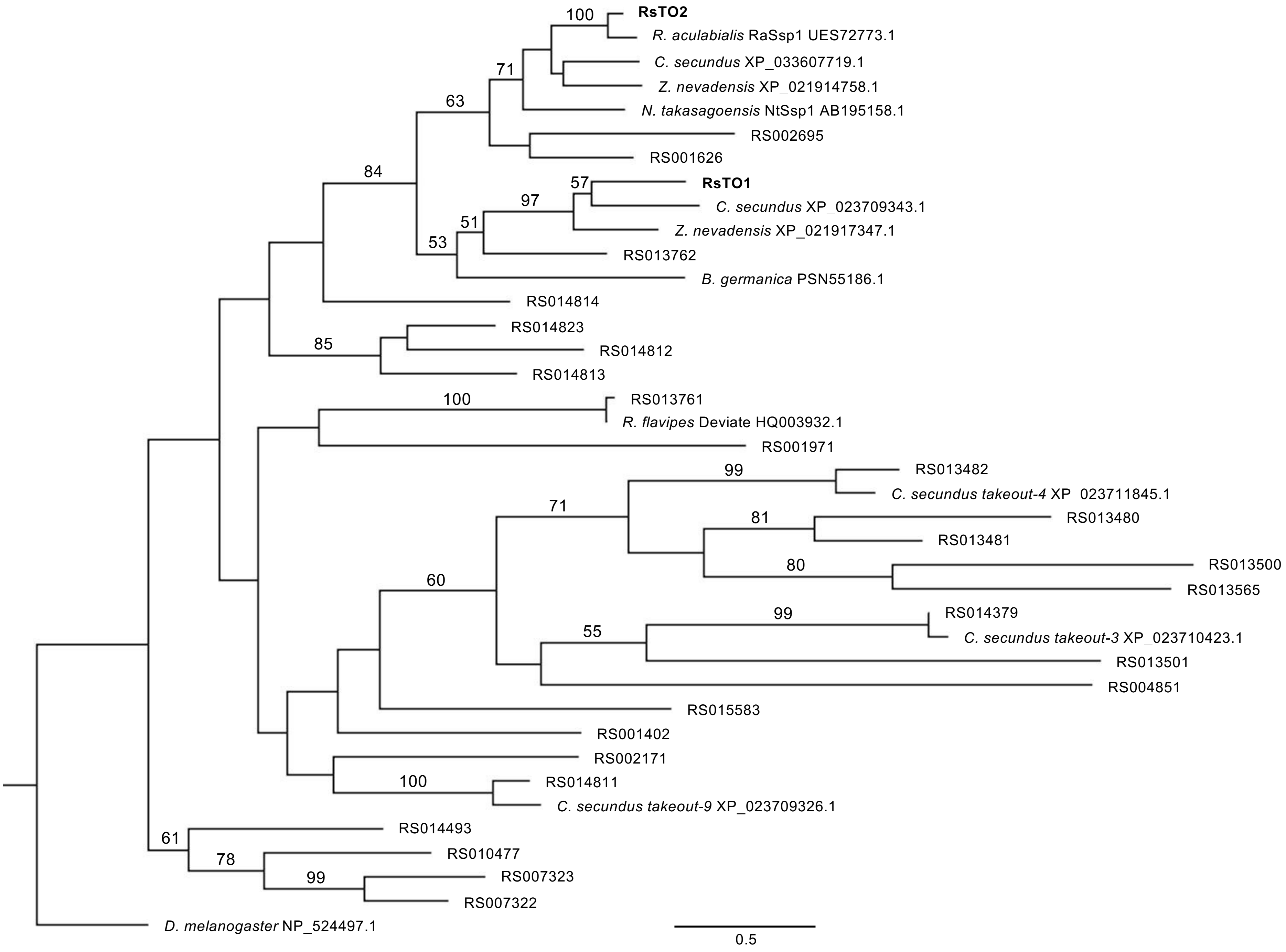
100
RsTO2
R. aculabialis RaSsp1 UES72773.1
C. secundus XP_033607719.1
71
Z. nevadensis XP_021914758.1
63
N. takasagoensis NtSsp1 AB195158.1
RS002695
RS001626
84
57
RsTO1
97
C. secundus XP_023709343.1
51
Z. nevadensis XP_021917347.1
53
RS013762
B. germanica PSN55186.1
RS014814
RS014823
85
RS014812
RS014813
100
RS013761
R. flavipes Deviate HQ003932.1
RS001971
99
RS013482
C. secundus takeout-4 XP_023711845.1
71
81
RS013480
RS013481
80
RS013500
60
RS013565
99
RS014379
55
C. secundus takeout-3 XP_023710423.1
RS013501
RS004851
RS015583
RS001402
RS002171
100
RS014811
C. secundus takeout-9 XP_023709326.1
RS014493
61
RS010477
78
99
RS007323
RS007322
D. melanogaster NP_524497.1
0.5

Fig. S2

Maximum likelihood (ML) tree of *takeout* homologs based on the amino acid sequences obtained with the LG+G4 model. Bootstrap probabilities of 100 ML trees (only >50%) are shown above or below each branch. The analysis involved 39 amino acid sequences, including *R. speratus takeout* genes (total of 27); *RsTO1* and *RsTO2* homologous genes of *Z. nevadensis*, *C. secundus* and *B. germanica* (total of 5); six *takeout* genes previously reported in termites (*RaSsp1*, *NtSsp1*, *Deviate*, *takeout-3*, *-4* and *-9*); and a *takeout* gene used for an outgroup (homolog of *Drosophila melanogaster*).

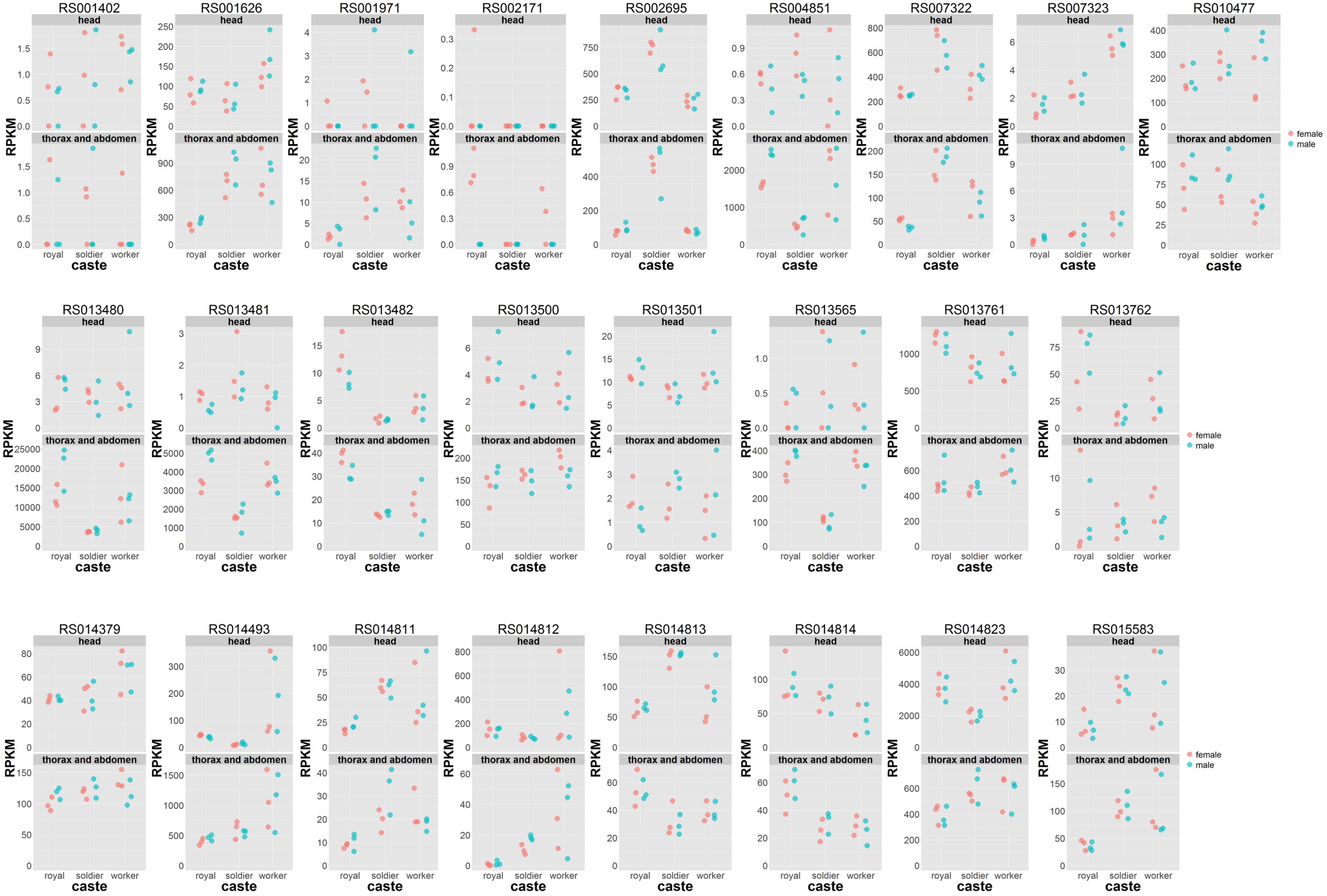
RS001402
RS001626
RS001971
RS002171
RS002695
RS004851
RS007322
RS007323
RS010477
RS013480
RS013481
RS013482
RS013500
RS013501
RS013565
RS013761
RS013762
RS014379
RS014493
RS014811
RS014812
RS014813
RS014814
RS014823
RS015583
head
thorax and abdomen
RPKM
royal
soldier
worker
caste
female
male

Fig. S3

Expression levels of 25 *takeout* genes among royals (reproductives), soldiers and workers in *R. speratus*. Expression levels are indicated as reads per kilobase million (RPKM) calculated from RNA-seq analysis (Shigenobu et al. 2022). Orange and blue points indicate females and males, respectively.

Table S1. Primer sequences used in this study.

Gene (accession no/gene ID)	Experiment	Sequence (5'->3')
RsTO1 (LC742508)	5'-RACE	CGCAGCCCATCGAAAAAGAGTTCGT
RsTO1 (LC742508)	3'-RACE	GCGGGATACACGCGACTTCCAAAAA
RsTO1 (LC742508)	qRT-PCR, forward	CCGACTACATCAAGCCGTGT
RsTO1 (LC742508)	qRT-PCR, reverse	CGCCATACCGAGAATCGTGA
RsTO1 (LC742508)	in situ hybridization, forward	GAGACGCCATACCGAGAATC
RsTO1 (LC742508)	in situ hybridization, reverse	AGGACGATACGCCAGTTGTC
RsTO2 (LC742509)	5'-RACE	GGGTACCGTCTTCGATGCGCCCGAGA
RsTO2 (LC742509)	3'-RACE	GGCGCGAAGTACTCAAGGTCGTTGG
RsTO2 (LC742509)	qRT-PCR, forward	AGTGCGAAGGAGACAGTTCC
RsTO2 (LC742509)	qRT-PCR, reverse	TTCAAGTCTCCATCGACCGC
RsTO2 (LC742509)	in situ hybridization, forward	CTGTACGTCACGGAGGTCAG
RsTO2 (LC742509)	in situ hybridization, reverse	GTGGACAACGAGACCCAGAG
GGPPS (RS100016)	qRT-PCR, forward	TGGGTGGAACGTTCATGTTTGT
GGPPS (RS100016)	qRT-PCR, reverse	CCGAATCTGGTAGTAGAGCCCC
RsVg1 (RS000616)	qRT-PCR, forward	CCTACATGCGTTGTTGATGG
RsVg1 (RS000616)	qRT-PCR, reverse	TGACGACTATGCACTCCAGC
RsVg2 (RS000610)	qRT-PCR, forward	GCGAAATGGAGAACCCTAAT
RsVg2 (RS000610)	qRT-PCR, reverse	TCACTTCGTCGATTGCTGTA
EF1-alfa (AB602838)	qRT-PCR, forward	GGTGATGCGGCTATTGTTAACC
EF1-alfa (AB602838)	qRT-PCR, reverse	GTGGTGGGAATTCTGAGAAAGATT
NADH-dh (AB602837)	qRT-PCR, forward	GCTGGGGGGTTATTCATTCCAT
NADH-dh (AB602837)	qRT-PCR, reverse	GGCATACCACAAAGGGCAAAA
beta-actin (AB520714)	qRT-PCR, forward	AGCGGGAAATCGTCCGTGAC
beta-actin (AB520714)	qRT-PCR, reverse	CAATGGTGATGACCTGCCCAT
GstD1 (RS001168)	qRT-PCR, forward	GCTGTTGGTGTGGATTTGAA
GstD1 (RS001168)	qRT-PCR, reverse	GTATGCTGCGGGTTCATCTT
EIF-1 (RS005199)	qRT-PCR, forward	ATGGTAGGCTTGAAGCGATG
EIF-1 (RS005199)	qRT-PCR, reverse	TTTGCATCCTGGTAGTCACG
RPS18 (RS015150)	qRT-PCR, forward	ACTCTCAGCTCACATCCAGT
RPS18 (RS015150)	qRT-PCR, reverse	CCTCAGGCCCCAATAATGTC

Table S2. *Takeout* genes identified in *Reticulitermes speratus*.

Gene ID[1]	Scaffold	Sequence[2]	Signal peptide[3]	Exon structure[4]
RS000936	1087	NTE	-	Huge gaps (>30k bases) between exons
LC742508 (named RsTO1)	1087	complete	0.5951	Newly identified in this study
LC742509 (named RsTO2)	1087	complete	0.9975	Newly identified in this study
RS001402	1187	NTE	-	OK
RS001624	1212	NTE	-	Single exon, too short (<200 bases)
RS001625	1212	CTE	0.9942	Huge gaps (>20k bases) between exons
RS001626	1212	NTE	-	OK
RS001971	1295	complete	None	OK
RS001972	1295	NTE	-	Single exon, too short (<200 bases)
RS002171	1316	complete	0.5936	OK
RS002692	1414	NTE	-	Single exon, too short (<200 bases)
RS002694	1414	fragment	-	Single exon, too short (<250 bases)
RS002695	1415	NTE	-	OK
RS004851	206	complete	0.996	OK
RS007322	302	complete	0.9798	OK
RS007323	302	complete	0.9053	OK
RS010477	478	complete	None	OK
RS013480	7	complete	0.9972	OK
RS013481	7	complete	0.9923	OK
RS013482	7	complete	0.8675	OK
RS013500	7	complete	0.9874	OK
RS013501	7	complete	0.9967	OK
RS013565	70	complete	0.9946	OK
RS013761	732	complete	0.9924	OK
RS013762	732	complete	0.9899	OK
RS014379	80	complete	0.9816	OK
RS014493	827	complete	0.9926	OK
RS014811	870	CTE	None	OK
RS014812	870	complete	0.9012	OK
RS014813	870	NTE	-	OK
RS014814	870	NTE	-	OK
RS014823	878	complete	0.9489	OK
RS015583	999	complete	0.9874	OK
RS015584	999	fragment	-	Single exon, too short (<200 bases)

[1]Shigenobu et al. (2022)

[2]NTE: initial methionine (M) is missed in the transcript sequence. CTE: stop codon is missed in the transcript sequence.

[3]Likelihood of signal peptide (Sec / SPI) calculated by SignalP - 5.0 :https://services.healthtech.dtu.dk/service.php?SignalP-5.0

[4]*R. speratus* genome browser: http://www.termite.nibb.info/retsp/

Table S3. Stability values of reference genes in qPCR analysis among castes.

Gene (accession no/gene ID)	Stability value	
	GeNorm	NormFinder
EF1-alfa (AB602838)	0.708	0.392
NADH-dh (AB602837)	0.574	0.101
beta-actin (AB520714)	1.099	0.741
GstD1 (RS001168)*	0.572	0.075
EIF-1 (RS005199)	0.556	0.132
RPS18 (RS015150)	0.725	0.424

*GstD1 was selected due to the stability values among six genes analyzed.

Table S4. Stability values of reference genes in qPCR analysis among female nymphs, alates, and queens.

Gene (accession no/gene ID)	Stability value	
	GeNorm	NormFinder
EF1-alfa (AB602838)	1.204	0.715
NADH-dh (AB602837)	1.199	0.634
beta-actin (AB520714)	1.471	0.927
GstD1 (RS001168)	0.948	0.26
EIF-1 (RS005199)*	0.85	0.136
RPS18 (RS015150)	0.993	0.48

**EIF-1* was selected due to the lowest stability values among 6 genes analyzed.

Table S5. Stability values of reference genes in qPCR analysis during soldier formation.

Gene (accession no/gene ID)	Stability value	
	GeNorm	NormFinder
EF1-alfa (AB602838)	0.924	0.536
NADH-dh (AB602837)	0.773	0.345
beta-actin (AB520714)	0.769	0.319
GstD1 (RS001168)	0.846	0.446
EIF-1 (RS005199)	0.938	0.562
RPS18 (RS015150)*	0.676	0.135

**RPS18* was selected due to the lowest stability values among 6 genes analyzed.

```
Selected Residue / Sequence
Deleted Residue / Sequence
                                                10        20        30        40        50        60
                                        =========+=========+=========+=========+=========+=========+
  RsTO1                                 ------------------------------------------------------------
  RsTO2                                 ------------------------------------------------------------
  RS001402                              ------------------------------------------------------------
  RS001626                              ------------------------------------------------------------
  RS001971                              ------------------------------------------------------------
  RS002171                              ------------------------------------------------------------
  RS002695                              ------------------------------------------------------------
  RS004851                              ------------------------------------------------------------
  RS007322                              ------------------------------------------------------------
  RS007323                              ------------------------------------------------------------
  RS010477                              MFK---------------DFYLPAAI----------------------------------
  RS013480                              ------------------------------------------------------------
  RS013481                              ------------------------------------------------------------
  RS013482                              M-----------------------------------------------------------
  RS013500                              ------------------------------------------------------------
  RS013501                              M-----------------------------------------------------------
  RS013565                              ------------------------------------------------------------
  RS013761                              M-----------------------------------------------------------
  RS013762                              MSA---------------------------------------------------------
  RS014379                              ------------------------------------------------------------
  RS014493                              M-----------------------------------------------------------
  RS014811                              ------------------------------------------------------------
  RS014812                              ML----------------------------------------------------------
  RS014813                              ------------------------------------------------------------
  RS014814                              ------------------------------------------------------------
  RS014823                              M-----------------------------------------------------------
  RS015583                              MTR-------------------P-------------------------------------
  Z.nevadensis_XP_021917347.1           ------------------------------------------------------------
  Z.nevadensis_XP_021924758.1           ------------------------------------------------------------
  C.secundus_XP_023709343.1             ------------------------------------------------------------
  C.secundus_XP_033607719.1             MSKLSIFFAVCAVVCQSEALKLPDYITPCSRKDPNFNECALKHGREAIPRLLKGDPKYGV
  C.secundus_takeout3_XP_023710423.1    ------------------------------------------------------------
  C.secundus_takeout4_XP_023711845.1    M-----------------------------------------------------------
  C.secundus_takeout9_XP_023709326.1    ------------------------------------------------------------
  B.germanica_PSN55186.1                ------------------------------------------------------------
  N.takasagoensis_NtSsp1_AB195158.1     ------------------------------------------------------------
  R.aculabialis_RaSsp1_UES72773.1       ------------------------------------------------------------
  R.flavipes__Deviate_HQ003932.1        M-----------------------------------------------------------
  D.melanogaster_NP_524497.1            MF----------------------------------------------------------

                                                70        80        90       100       110       120
                                        =========+=========+=========+=========+=========+=========+
  RsTO1                                 ------------------------------------------------------------
  RsTO2                                 ------------------------------------------------------------
  RS001402                              ------------------------------------------------------------
  RS001626                              ------------------------------------------------------------
  RS001971                              ------------------------------------------------------------
  RS002171                              ------------------------------------------------------------
  RS002695                              ------------------------------------------------------------
  RS004851                              ------------------------------------------------------------
  RS007322                              ------------------------------------------------------------
  RS007323                              ------------------------------------------------------------
  RS010477                              ------------------------------------------------------------
  RS013480                              ------------------------------------------------------------
  RS013481                              ------------------------------------------------------------
  RS013482                              ------------------------------------------------------------
  RS013500                              ------------------------------------------------------------
  RS013501                              ------------------------------------------------------------
  RS013565                              ------------------------------------------------------------
  RS013761                              ------------------------------------------------------------
  RS013762                              ------------------------------------------------------------
  RS014379                              ------------------------------------------------------------
  RS014493                              ------------------------------------------------------------
  RS014811                              ------------------------------------------------------------
  RS014812                              ------------------------------------------------------------
  RS014813                              ------------------------------------------------------------
  RS014814                              ------------------------------------------------------------
  RS014823                              ------------------------------------------------------------
  RS015583                              ------------------------------------------------------------
  Z.nevadensis_XP_021917347.1           ------------------------------------------------------------
```

```
Z.nevadensis_XP_021924758.1             ------------------------------------------------------------
C.secundus_XP_023709343.1               ------------------------------------------------------------
C.secundus_XP_033607719.1               PVLDPMYVPEVSVEESGIKITARNFTIEGARNATLQDFRVDFDKLIVSIQFMAPQLDFRG
C.secundus_takeout3_XP_023710423.1      ------------------------------------------------------------
C.secundus_takeout4_XP_023711845.1      ------------------------------------------------------------
C.secundus_takeout9_XP_023709326.1      ------------------------------------------------------------
B.germanica_PSN55186.1                  ------------------------------------------------------------
N.takasagoensis_NtSsp1_AB195158.1       ------------------------------------------------------------
R.aculabialis_RaSsp1_UES72773.1         ------------------------------------------------------------
R.flavipes__Deviate_HQ003932.1          ------------------------------------------------------------
D.melanogaster_NP_524497.1              ------------------------------------------------------------

                                               130       140       150       160       170       180
                                        =========+=========+=========+=========+=========+=========+
RsTO1                                   ------------------------------------------------------------
RsTO2                                   ------------------------------------------------------------
RS001402                                ------------------------------------------------------------
RS001626                                ------------------------------------------------------------
RS001971                                ------------------------------------------------------------
RS002171                                ------------------------------------------------------------
RS002695                                ------------------------------------------------------------
RS004851                                ------------------------------------------------------------
RS007322                                ------------------------------------------------------------
RS007323                                ------------------------------------------------------------
RS010477                                ------------------------------------------------------------
RS013480                                ------------------------------------------------------------
RS013481                                ------------------------------------------------------------
RS013482                                ------------------------------------------------------------
RS013500                                ------------------------------------------------------------
RS013501                                ------------------------------------------------------------
RS013565                                ------------------------------------------------------------
RS013761                                ------------------------------------------------------------
RS013762                                ------------------------------------------------------------
RS014379                                ------------------------------------------------------------
RS014493                                ------------------------------------------------------------
RS014811                                ------------------------------------------------------------
RS014812                                ------------------------------------------------------------
RS014813                                ------------------------------------------------------------
RS014814                                ------------------------------------------------------------
RS014823                                ------------------------------------------------------------
RS015583                                ------------------------------------------------------------
Z.nevadensis_XP_021917347.1             ------------------------------------------------------------
Z.nevadensis_XP_021924758.1             ------------------------------------------------------------
C.secundus_XP_023709343.1               ------------------------------------------------------------
C.secundus_XP_033607719.1               KYELSGKLVSLPIIGKGDFNSTFRGLIAKYETQCNFTKKADGKTYLMPLDYEMEFEPEFV
C.secundus_takeout3_XP_023710423.1      ------------------------------------------------------------
C.secundus_takeout4_XP_023711845.1      ------------------------------------------------------------
C.secundus_takeout9_XP_023709326.1      ------------------------------------------------------------
B.germanica_PSN55186.1                  ------------------------------------------------------------
N.takasagoensis_NtSsp1_AB195158.1       ------------------------------------------------------------
R.aculabialis_RaSsp1_UES72773.1         ------------------------------------------------------------
R.flavipes__Deviate_HQ003932.1          ------------------------------------------------------------
D.melanogaster_NP_524497.1              ------------------------------------------------------------

                                               190       200       210       220       230       240
                                        =========+=========+=========+=========+=========+=========+
RsTO1                                   ------------------------------------------------------------
RsTO2                                   ------------------------------------------------------------
RS001402                                ------------------------------------------------------------
RS001626                                ------------------------------------------------------------
RS001971                                ------------------------------------------------------------
RS002171                                ------------------------------------------------------------
RS002695                                ------------------------------------------------------------
RS004851                                ------------------------------------------------------------
RS007322                                ------------------------------------------------------------
RS007323                                ------------------------------------------------------------
RS010477                                ------LF----------------------------------------------------
RS013480                                ------------------------------------------------------------
RS013481                                ------------------------------------------------------------
RS013482                                ------------------------------------------------------------
RS013500                                ------------------------------------------------------------
RS013501                                ------------------------------------------------------------
RS013565                                ------------------------------------------------------------
RS013761                                ------------------------------------------------------------
RS013762                                ------------------------------------------------------------
```

```
RS014379                                ------------------------------------------------------------
RS014493                                ------------------------------------------------------------
RS014811                                ------------------------------------------------------------
RS014812                                ------------------------------------------------------------
RS014813                                ------------------------------------------------------------
RS014814                                ------------------------------------------------------------
RS014823                                ------------------------------------------------------------
RS015583                                ------------------------------------------------------------
Z.nevadensis_XP_021917347.1             ------------------------------------------------------------
Z.nevadensis_XP_021924758.1             ------------------------------------------------------------
C.secundus_XP_023709343.1               ------------------------------------------------------------
C.secundus_XP_033607719.1               RIYFGNLFNGNKLLGDAMNSFIAENWRLVLDQIGKPAYRALGMIVHQILVQVANKVPYDE
C.secundus_takeout3_XP_023710423.1      ------------------------------------------------------------
C.secundus_takeout4_XP_023711845.1      ------------------------------------------------------------
C.secundus_takeout9_XP_023709326.1      ------------------------------------------------------------
B.germanica_PSN55186.1                  ------------------------------------------------------------
N.takasagoensis_NtSsp1_AB195158.1       ------------------------------------------------------------
R.aculabialis_RaSsp1_UES72773.1         ------------------------------------------------------------
R.flavipes__Deviate_HQ003932.1          ------------------------------------------------------------
D.melanogaster_NP_524497.1              ------------------------------------------------------------

                                               250       260       270       280       290       300
                                        =========+=========+=========+=========+=========+=========+
RsTO1                                   ------------------------------------------------------------
RsTO2                                   ------------------------------------------------------------
RS001402                                ------------------------------------------------------------
RS001626                                ------------------------------------------------------------
RS001971                                ------------------------------------------------------------
RS002171                                ------------------------------------------------------------
RS002695                                ------------------------------------------------------------
RS004851                                ------------------------------------------------------------
RS007322                                ------------------------------------------------------------
RS007323                                ------------------------------------------------------------
RS010477                                ------------------------------------------------------------
RS013480                                ------------------------------------------------------------
RS013481                                ------------------------------------------------------------
RS013482                                ------------------------------------------------------------
RS013500                                ------------------------------------------------------------
RS013501                                ------------------------------------------------------------
RS013565                                ------------------------------------------------------------
RS013761                                ------------------------------------------------------------
RS013762                                ------------------------------------------------------------
RS014379                                ------------------------------------------------------------
RS014493                                ------------------------------------------------------------
RS014811                                ------------------------------------------------------------
RS014812                                ------------------------------------------------------------
RS014813                                ------------------------------------------------------------
RS014814                                ------------------------------------------------------------
RS014823                                ------------------------------------------------------------
RS015583                                ------------------------------------------------------------
Z.nevadensis_XP_021917347.1             ------------------------------------------------------------
Z.nevadensis_XP_021924758.1             ------------------------------------------------------------
C.secundus_XP_023709343.1               ------------------------------------------------------------
C.secundus_XP_033607719.1               LFSDTDTVSGSWDLLISRRLCISGDPKYGIPVLDPMYIPEVSIQESGIKITGREIVIEGA
C.secundus_takeout3_XP_023710423.1      ------------------------------------------------------------
C.secundus_takeout4_XP_023711845.1      ------------------------------------------------------------
C.secundus_takeout9_XP_023709326.1      ------------------------------------------------------------
B.germanica_PSN55186.1                  ------------------------------------------------------------
N.takasagoensis_NtSsp1_AB195158.1       ------------------------------------------------------------
R.aculabialis_RaSsp1_UES72773.1         ------------------------------------------------------------
R.flavipes__Deviate_HQ003932.1          ------------------------------------------------------------
D.melanogaster_NP_524497.1              ------------------------------------------------------------

                                               310       320       330       340       350       360
                                        =========+=========+=========+=========+=========+=========+
RsTO1                                   ------------------------------------------------------------
RsTO2                                   ------------------------------------------------------------
RS001402                                ------------------------------------------------------------
RS001626                                ------------------------------------------------------------
RS001971                                ------------------------------------------------------------
RS002171                                ------------------------------------------------------------
RS002695                                ------------------------------------------------------------
RS004851                                ------------------------------------------------------------
RS007322                                ------------------------------------------------------------
RS007323                                ------------------------------------------------------------
```

```
RS010477                               ------------------------------------------------------------
RS013480                               ------------------------------------------------------------
RS013481                               ------------------------------------------------------------
RS013482                               ------------------------------------------------------------
RS013500                               ------------------------------------------------------------
RS013501                               ------------------------------------------------------------
RS013565                               ------------------------------------------------------------
RS013761                               ------------------------------------------------------------
RS013762                               ------------------------------------------------------------
RS014379                               ------------------------------------------------------------
RS014493                               ------------------------------------------------------------
RS014811                               ------------------------------------------------------------
RS014812                               ------------------------------------------------------------
RS014813                               ------------------------------------------------------------
RS014814                               ------------------------------------------------------------
RS014823                               ------------------------------------------------------------
RS015583                               ------------------------------------------------------------
Z.nevadensis_XP_021917347.1            ------------------------------------------------------------
Z.nevadensis_XP_021924758.1            ------------------------------------------------------------
C.secundus_XP_023709343.1              ------------------------------------------------------------
C.secundus_XP_033607719.1              RNAILEDFRVDFDKLIVSIQFMAPQLDFRGKYELSGKLVSLPIVGKGDYNATLGNLTAKY
C.secundus_takeout3_XP_023710423.1     ------------------------------------------------------------
C.secundus_takeout4_XP_023711845.1     ------------------------------------------------------------
C.secundus_takeout9_XP_023709326.1     ------------------------------------------------------------
B.germanica_PSN55186.1                 ------------------------------------------------------------
N.takasagoensis_NtSsp1_AB195158.1      ------------------------------------------------------------
R.aculabialis_RaSsp1_UES72773.1        ------------------------------------------------------------
R.flavipes__Deviate_HQ003932.1         ------------------------------------------------------------
D.melanogaster_NP_524497.1             ------------------------------------------------------------

                                              370       380       390       400       410       420
                                       =========+=========+=========+=========+=========+=========+
RsTO1                                  ------------------------------------------------------------
RsTO2                                  ------------------------------------------------------------
RS001402                               ------------------------------------------------------------
RS001626                               ------------------------------------------------------------
RS001971                               ------------------------------------------------------------
RS002171                               ------------------------------------------------------------
RS002695                               ------------------------------------------------------------
RS004851                               ------------------------------------------------------------
RS007322                               ------------------------------------------------------------
RS007323                               ------------------------------------------------------------
RS010477                               --QCD---------------------------------------------C---------
RS013480                               ------------------------------------------------------------
RS013481                               ------------------------------------------------------------
RS013482                               ------------------------------------------------------------
RS013500                               ------------------------------------------------------------
RS013501                               ------------------------------------------------------------
RS013565                               ------------------------------------------------------------
RS013761                               ------------------------------------------------------------
RS013762                               ------------------------------------------------------------
RS014379                               ------------------------------------------------------------
RS014493                               ------------------------------------------------------------
RS014811                               ------------------------------------------------------------
RS014812                               ------------------------------------------------------------
RS014813                               ------------------------------------------------------------
RS014814                               ------------------------------------------------------------
RS014823                               ------------------------------------------------------------
RS015583                               ------------------------------------------------------------
Z.nevadensis_XP_021917347.1            ------------------------------------------------------------
Z.nevadensis_XP_021924758.1            ------------------------------------------------------------
C.secundus_XP_023709343.1              ------------------------------------------------------------
C.secundus_XP_033607719.1              TTQCNLTKQGDGRTYLSPQDYDLEFDPKTASMYFGNLFNGNKLLADYMKPCSRSDPNFNA
C.secundus_takeout3_XP_023710423.1     ------------------------------------------------------------
C.secundus_takeout4_XP_023711845.1     ------------------------------------------------------------
C.secundus_takeout9_XP_023709326.1     ------------------------------------------------------------
B.germanica_PSN55186.1                 ------------------------------------------------------------
N.takasagoensis_NtSsp1_AB195158.1      ------------------------------------------------------------
R.aculabialis_RaSsp1_UES72773.1        ------------------------------------------------------------
R.flavipes__Deviate_HQ003932.1         ------------------------------------------------------------
D.melanogaster_NP_524497.1             ------------------------------------------------------------

                                              430       440       450       460       470       480
                                       =========+=========+=========+=========+=========+=========+
RsTO1                                  ------------------------------------------------------------
```

```
RsTO2                                      ------------------------------------------------------------
RS001402                                   ------------------------------------------------------------
RS001626                                   ------------------------------------------------------------
RS001971                                   ------------------------------------------------------------
RS002171                                   ------------------------------------------------------------
RS002695                                   ------------------------------------------------------------
RS004851                                   ------------------------------------------------------------
RS007322                                   ------------------------------------------------------------
RS007323                                   ------------------------------------------------------------
RS010477                                   ------------------------------------------------------------
RS013480                                   ------------------------------------------------------------
RS013481                                   ------------------------------------------------------------
RS013482                                   ------------------------------------------------------------
RS013500                                   ------------------------------------------------------------
RS013501                                   ------------------------------------------------------------
RS013565                                   ------------------------------------------------------------
RS013761                                   ------------------------------------------------------------
RS013762                                   ------------------------------------------------------------
RS014379                                   ------------------------------------------------------------
RS014493                                   ------------------------------------------------------------
RS014811                                   ------------------------------------------------------------
RS014812                                   ------------------------------------------------------------
RS014813                                   ------------------------------------------------------------
RS014814                                   ------------------------------------------------------------
RS014823                                   ------------------------------------------------------------
RS015583                                   ------------------------------------------------------------
Z.nevadensis_XP_021917347.1                ------------------------------------------------------------
Z.nevadensis_XP_021924758.1                ------------------------------------------------------------
C.secundus_XP_023709343.1                  ------------------------------------------------------------
C.secundus_XP_033607719.1                  CALQQGREAMSRMVQGDDKYGVPVLDPLLIRKLTTTYNGFTATTRNSTVVGVKGVVIEDI
C.secundus_takeout3_XP_023710423.1         ------------------------------------------------------------
C.secundus_takeout4_XP_023711845.1         ------------------------------------------------------------
C.secundus_takeout9_XP_023709326.1         ------------------------------------------------------------
B.germanica_PSN55186.1                     ------------------------------------------------------------
N.takasagoensis_NtSsp1_AB195158.1          ------------------------------------------------------------
R.aculabialis_RaSsp1_UES72773.1            ------------------------------------------------------------
R.flavipes__Deviate_HQ003932.1             ------------------------------------------------------------
D.melanogaster_NP_524497.1                 ------------------------------------------------------------

                                                  490       500       510       520       530       540
                                           =========+=========+=========+=========+=========+=========+
RsTO1                                      ------------------------------------------------------------
RsTO2                                      ------------------------------------------------------------
RS001402                                   ------------------------------------------------------------
RS001626                                   ------------------------------------------------------------
RS001971                                   ------------------------------------------------------------
RS002171                                   ------------------------------------------------------------
RS002695                                   ------------------------------------------------------------
RS004851                                   ------------------------------------------------------------
RS007322                                   ------------------------------------------------------------
RS007323                                   ------------------------------------------------------------
RS010477                                   ------------------------------------------------------------
RS013480                                   ------------------------------------------------------------
RS013481                                   ------------------------------------------------------------
RS013482                                   ------------------------------------------------------------
RS013500                                   ------------------------------------------------------------
RS013501                                   ------------------------------------------------------------
RS013565                                   ------------------------------------------------------------
RS013761                                   ------------------------------------------------------------
RS013762                                   ------------------------------------------------------------
RS014379                                   ------------------------------------------------------------
RS014493                                   ------------------------------------------------------------
RS014811                                   ------------------------------------------------------------
RS014812                                   ------------------------------------------------------------
RS014813                                   ------------------------------------------------------------
RS014814                                   ------------------------------------------------------------
RS014823                                   ------------------------------------------------------------
RS015583                                   ------------------------------------------------------------
Z.nevadensis_XP_021917347.1                ------------------------------------------------------------
Z.nevadensis_XP_021924758.1                ------------------------------------------------------------
C.secundus_XP_023709343.1                  ------------------------------------------------------------
C.secundus_XP_033607719.1                  RPQTCPMGVLHKYTNAYGRVNLNLHISKQSFGTFRFLVHSFDFDKQLVRVAFMFPTLQFN
C.secundus_takeout3_XP_023710423.1         ------------------------------------------------------------
C.secundus_takeout4_XP_023711845.1         ------------------------------------------------------------
C.secundus_takeout9_XP_023709326.1         ------------------------------------------------------------
```

```
B.germanica_PSN55186.1                    ------------------------------------------------------------
N.takasagoensis_NtSsp1_AB195158.1         ------------------------------------------------------------
R.aculabialis_RaSsp1_UES72773.1           ------------------------------------------------------------
R.flavipes__Deviate_HQ003932.1            ------------------------------------------------------------
D.melanogaster_NP_524497.1                ------------------------------------------------------------

                                                 550       560       570       580       590       600
                                          =========+=========+=========+=========+=========+=========+
RsTO1                                     ------------------------------------------------------------
RsTO2                                     ------------------------------------------------------------
RS001402                                  ------------------------------------------------------------
RS001626                                  ------------------------------------------------------------
RS001971                                  ------------------------------------------------------------
RS002171                                  ------------------------------------------------------------
RS002695                                  ------------------------------------------------------------
RS004851                                  ------------------------------------------------------------
RS007322                                  ------------------------------------------------------------
RS007323                                  ------------------------------------------------------------
RS010477                                  ------------------------------------------------------------
RS013480                                  ------------------------------------------------------------
RS013481                                  ------------------------------------------------------------
RS013482                                  ------------------------------------------------------------
RS013500                                  ------------------------------------------------------------
RS013501                                  ------------------------------------------------------------
RS013565                                  ------------------------------------------------------------
RS013761                                  ------------------------------------------------------------
RS013762                                  ------------------------------------------------------------
RS014379                                  ------------------------------------------------------------
RS014493                                  ------------------------------------------------------------
RS014811                                  ------------------------------------------------------------
RS014812                                  ------------------------------------------------------------
RS014813                                  ------------------------------------------------------------
RS014814                                  ------------------------------------------------------------
RS014823                                  ------------------------------------------------------------
RS015583                                  ------------------------------------------------------------
Z.nevadensis_XP_021917347.1               ------------------------------------------------------------
Z.nevadensis_XP_021924758.1               ------------------------------------------------------------
C.secundus_XP_023709343.1                 ------------------------------------------------------------
C.secundus_XP_033607719.1                 GLYEMSGKLAGLPISGKGDYDLFLRLKKFHIPVLDPLIVKEIRAVEGQLNMTGLDIKAEG
C.secundus_takeout3_XP_023710423.1        ------------------------------------------------------------
C.secundus_takeout4_XP_023711845.1        ------------------------------------------------------------
C.secundus_takeout9_XP_023709326.1        ------------------------------------------------------------
B.germanica_PSN55186.1                    ------------------------------------------------------------
N.takasagoensis_NtSsp1_AB195158.1         ------------------------------------------------------------
R.aculabialis_RaSsp1_UES72773.1           ------------------------------------------------------------
R.flavipes__Deviate_HQ003932.1            ------------------------------------------------------------
D.melanogaster_NP_524497.1                ------------------------------------------------------------

                                                 610       620       630       640       650       660
                                          =========+=========+=========+=========+=========+=========+
RsTO1                                     ------------------------------------------------------------
RsTO2                                     ------------------------------------------------------------
RS001402                                  ------------------------------------------------------------
RS001626                                  ------------------------------------------------------------
RS001971                                  ------------------------------------------------------------
RS002171                                  ------------------------------------------------------------
RS002695                                  ------------------------------------------------------------
RS004851                                  ------------------------------------------------------------
RS007322                                  ------------------------------------------------------------
RS007323                                  ------------------------------------------------------------
RS010477                                  ------------------------------------------------------------
RS013480                                  ------------------------------------------------------------
RS013481                                  ------------------------------------------------------------
RS013482                                  ------------------------------------------------------------
RS013500                                  ------------------------------------------------------------
RS013501                                  ------------------------------------------------------------
RS013565                                  ------------------------------------------------------------
RS013761                                  ------------------------------------------------------------
RS013762                                  ------------------------------------------------------------
RS014379                                  ------------------------------------------------------------
RS014493                                  ------------------------------------------------------------
RS014811                                  ------------------------------------------------------------
RS014812                                  ------------------------------------------------------------
RS014813                                  ------------------------------------------------------------
RS014814                                  ------------------------------------------------------------
```

```
RS014823                                ------------------------------------------------------------
RS015583                                ------------------------------------------------------------
Z.nevadensis_XP_021917347.1             ------------------------------------------------------------
Z.nevadensis_XP_021924758.1             ------------------------------------------------------------
C.secundus_XP_023709343.1               ------------------------------------------------------------
C.secundus_XP_033607719.1               MRKAILESISFDFDQKKVVMEVVVPEAHFSGRYEVSGKLGALPIKGKGTLDATFYHLYVK
C.secundus_takeout3_XP_023710423.1      ------------------------------------------------------------
C.secundus_takeout4_XP_023711845.1      ------------------------------------------------------------
C.secundus_takeout9_XP_023709326.1      ------------------------------------------------------------
B.germanica_PSN55186.1                  ------------------------------------------------------------
N.takasagoensis_NtSsp1_AB195158.1       ------------------------------------------------------------
R.aculabialis_RaSsp1_UES72773.1         ------------------------------------------------------------
R.flavipes__Deviate_HQ003932.1          ------------------------------------------------------------
D.melanogaster_NP_524497.1              ------------------------------------------------------------

                                               670       680       690       700       710       720
                                        =========+=========+=========+=========+=========+=========+
RsTO1                                   ------------------------------------------------------------
RsTO2                                   ------------------------------------------------------------
RS001402                                ------------------------------------------------------------
RS001626                                ------------------------------------------------------------
RS001971                                ------------------------------------------------------------
RS002171                                ------------------------------------------------------------
RS002695                                ------------------------------------------------------------
RS004851                                ------------------------------------------------------------
RS007322                                ------------------------------------------------------------
RS007323                                ------------------------------------------------------------
RS010477                                ------------------------------------------------------------
RS013480                                ------------------------------------------------------------
RS013481                                ------------------------------------------------------------
RS013482                                ------------------------------------------------------------
RS013500                                ------------------------------------------------------------
RS013501                                ------------------------------------------------------------
RS013565                                ------------------------------------------------------------
RS013761                                ------------------------------------------------------------
RS013762                                ------------------------------------------------------------
RS014379                                ------------------------------------------------------------
RS014493                                ------------------------------------------------------------
RS014811                                ------------------------------------------------------------
RS014812                                ------------------------------------------------------------
RS014813                                ------------------------------------------------------------
RS014814                                ------------------------------------------------------------
RS014823                                ------------------------------------------------------------
RS015583                                ------------------------------------------------------------
Z.nevadensis_XP_021917347.1             ------------------------------------------------------------
Z.nevadensis_XP_021924758.1             ------------------------------------------------------------
C.secundus_XP_023709343.1               ------------------------------------------------------------
C.secundus_XP_033607719.1               YVTTFDLTKHADGQVYLEPKKYKVEFDAKNMKAHLGNLFNGNKVLGDTMNKFINENWRTL
C.secundus_takeout3_XP_023710423.1      ------------------------------------------------------------
C.secundus_takeout4_XP_023711845.1      ------------------------------------------------------------
C.secundus_takeout9_XP_023709326.1      ------------------------------------------------------------
B.germanica_PSN55186.1                  ------------------------------------------------------------
N.takasagoensis_NtSsp1_AB195158.1       ------------------------------------------------------------
R.aculabialis_RaSsp1_UES72773.1         ------------------------------------------------------------
R.flavipes__Deviate_HQ003932.1          ------------------------------------------------------------
D.melanogaster_NP_524497.1              ------------------------------------------------------------

                                               730       740       750       760       770       780
                                        =========+=========+=========+=========+=========+=========+
RsTO1                                   ------------------------------------------------------------
RsTO2                                   ------------------------------------------------------------
RS001402                                ------------------------------------------------------------
RS001626                                ------------------------------------------------------------
RS001971                                ------------------------------------------------------------
RS002171                                ------------------------------------------------------------
RS002695                                ------------------------------------------------------------
RS004851                                ------------------------------------------------------------
RS007322                                ------------------------------------------------------------
RS007323                                ------------------------------------------------------------
RS010477                                ------------------------------------------------------------
RS013480                                ------------------------------------------------------------
RS013481                                ------------------------------------------------------------
RS013482                                ------------------------------------------------------------
RS013500                                ------------------------------------------------------------
RS013501                                ------------------------------------------------------------
```

```
RS013565                                    ------------------------------------------------------------
RS013761                                    ------------------------------------------------------------
RS013762                                    ------------------------------------------------------------
RS014379                                    ------------------------------------------------------------
RS014493                                    ------------------------------------------------------------
RS014811                                    ------------------------------------------------------------
RS014812                                    ------------------------------------------------------------
RS014813                                    ------------------------------------------------------------
RS014814                                    ------------------------------------------------------------
RS014823                                    ------------------------------------------------------------
RS015583                                    ------------------------------------------------------------
Z.nevadensis_XP_021917347.1                 ------------------------------------------------------------
Z.nevadensis_XP_021924758.1                 ------------------------------------------------------------
C.secundus_XP_023709343.1                   ------------------------------------------------------------
C.secundus_XP_033607719.1                   KKELGQPTYDALGSIAHKILSDAARMLGSSVTSQSHKREIEMEKTLALLVTCCLLLSATA
C.secundus_takeout3_XP_023710423.1          ------------------------------------------------------------
C.secundus_takeout4_XP_023711845.1          ------------------------------------------------------------
C.secundus_takeout9_XP_023709326.1          ------------------------------------------------------------
B.germanica_PSN55186.1                      ------------------------------------------------------------
N.takasagoensis_NtSsp1_AB195158.1           ------------------------------------------------------------
R.aculabialis_RaSsp1_UES72773.1             ------------------------------------------------------------
R.flavipes__Deviate_HQ003932.1              ------------------------------------------------------------
D.melanogaster_NP_524497.1                  ------------------------------------------------------------

                                                   790       800       810       820       830       840
                                            =========+=========+=========+=========+=========+=========+
RsTO1                                       ------------------------------------------------------------
RsTO2                                       ------------------------------------------------------------
RS001402                                    ------------------------------------------------------------
RS001626                                    ------------------------------------------------------------
RS001971                                    ------------------------------------------------------------
RS002171                                    ------------------------------------------------------------
RS002695                                    ------------------------------------------------------------
RS004851                                    ------------------------------------------------------------
RS007322                                    ------------------------------------------------------------
RS007323                                    ------------------------------------------------------------
RS010477                                    ------------------------------------------------------------
RS013480                                    ------------------------------------------------------------
RS013481                                    ------------------------------------------------------------
RS013482                                    ------------------------------------------------------------
RS013500                                    ------------------------------------------------------------
RS013501                                    ------------------------------------------------------------
RS013565                                    ------------------------------------------------------------
RS013761                                    ------------------------------------------------------------
RS013762                                    ------------------------------------------------------------
RS014379                                    ------------------------------------------------------------
RS014493                                    ------------------------------------------------------------
RS014811                                    ------------------------------------------------------------
RS014812                                    ------------------------------------------------------------
RS014813                                    ------------------------------------------------------------
RS014814                                    ------------------------------------------------------------
RS014823                                    ------------------------------------------------------------
RS015583                                    ------------------------------------------------------------
Z.nevadensis_XP_021917347.1                 ------------------------------------------------------------
Z.nevadensis_XP_021924758.1                 ------------------------------------------------------------
C.secundus_XP_023709343.1                   ------------------------------------------------------------
C.secundus_XP_033607719.1                   LKLPDYIKKCSIKDPNFDACALKNAKESLPNLITGDTEHKIPVLDPLFVEELRVDAKTIN
C.secundus_takeout3_XP_023710423.1          ------------------------------------------------------------
C.secundus_takeout4_XP_023711845.1          ------------------------------------------------------------
C.secundus_takeout9_XP_023709326.1          ------------------------------------------------------------
B.germanica_PSN55186.1                      ------------------------------------------------------------
N.takasagoensis_NtSsp1_AB195158.1           ------------------------------------------------------------
R.aculabialis_RaSsp1_UES72773.1             ------------------------------------------------------------
R.flavipes__Deviate_HQ003932.1              ------------------------------------------------------------
D.melanogaster_NP_524497.1                  ------------------------------------------------------------

                                                   850       860       870       880       890       900
                                            =========+=========+=========+=========+=========+=========+
RsTO1                                       ------------------------------------------------------------
RsTO2                                       ------------------------------------------------------------
RS001402                                    ------------------------------------------------------------
RS001626                                    ------------------------------------------------------------
RS001971                                    ------------------------------------------------------------
RS002171                                    ------------------------------------------------------------
RS002695                                    ------------------------------------------------------------
```

```
RS004851                                ------------------------------------------------------------
RS007322                                ------------------------------------------------------------
RS007323                                ------------------------------------------------------------
RS010477                                ------------------------------------------------------------
RS013480                                ------------------------------------------------------------
RS013481                                ------------------------------------------------------------
RS013482                                ------------------------------------------------------------
RS013500                                ------------------------------------------------------------
RS013501                                ------------------------------------------------------------
RS013565                                ------------------------------------------------------------
RS013761                                ------------------------------------------------------------
RS013762                                ------------------------------------------------------------
RS014379                                ------------------------------------------------------------
RS014493                                ------------------------------------------------------------
RS014811                                ------------------------------------------------------------
RS014812                                ------------------------------------------------------------
RS014813                                ------------------------------------------------------------
RS014814                                ------------------------------------------------------------
RS014823                                ------------------------------------------------------------
RS015583                                ------------------------------------------------------------
Z.nevadensis_XP_021917347.1             ------------------------------------------------------------
Z.nevadensis_XP_021924758.1             ------------------------------------------------------------
C.secundus_XP_023709343.1               ------------------------------------------------------------
C.secundus_XP_033607719.1               SSLRRATVTGLKIVSLKSVRFDFDKKSIAVEAVLPVLNFTGDYEVKGKLIGIPIYGNGPM
C.secundus_takeout3_XP_023710423.1      ------------------------------------------------------------
C.secundus_takeout4_XP_023711845.1      ------------------------------------------------------------
C.secundus_takeout9_XP_023709326.1      ------------------------------------------------------------
B.germanica_PSN55186.1                  ------------------------------------------------------------
N.takasagoensis_NtSsp1_AB195158.1       ------------------------------------------------------------
R.aculabialis_RaSsp1_UES72773.1         ------------------------------------------------------------
R.flavipes__Deviate_HQ003932.1          ------------------------------------------------------------
D.melanogaster_NP_524497.1              ------------------------------------------------------------

                                               910       920       930       940       950       960
                                        =========+=========+=========+=========+=========+=========+
RsTO1                                   ------------------------------------------------------------
RsTO2                                   ------------------------------------------------------------
RS001402                                ------------------------------------------------------------
RS001626                                ------------------------------------------------------------
RS001971                                ------------------------------------------------------------
RS002171                                ------------------------------------------------------------
RS002695                                ------------------------------------------------------------
RS004851                                ------------------------------------------------------------
RS007322                                ------------------------------------------------------------
RS007323                                ------------------------------------------------------------
RS010477                                ----------YKT----------EIFFA--------------------------------
RS013480                                ------------------------------------------------------------
RS013481                                ------------------------------------------------------------
RS013482                                ------------------------------------------------------------
RS013500                                ------------------------------------------------------------
RS013501                                ------------------------------------------------------------
RS013565                                ------------------------------------------------------------
RS013761                                ------------------------------------------------------------
RS013762                                ------------------------------------------------------------
RS014379                                ------------------------------------------------------------
RS014493                                ------------------------------------------------------------
RS014811                                ------------------------------------------------------------
RS014812                                ------------------------------------------------------------
RS014813                                ------------------------------------------------------------
RS014814                                ------------------------------------------------------------
RS014823                                ------------------------------------------------------------
RS015583                                ------------------------------------------------------------
Z.nevadensis_XP_021917347.1             ------------------------------------------------------------
Z.nevadensis_XP_021924758.1             ------------------------------------------------------------
C.secundus_XP_023709343.1               ------------------------------------------------------------
C.secundus_XP_033607719.1               NASLYNTKGNYRTTYDLAKLDDGEVYLVLQDHRLTVDPAHATVELGNLFGGNEVLGYYVN
C.secundus_takeout3_XP_023710423.1      ------------------------------------------------------------
C.secundus_takeout4_XP_023711845.1      ------------------------------------------------------------
C.secundus_takeout9_XP_023709326.1      ------------------------------------------------------------
B.germanica_PSN55186.1                  ------------------------------------------------------------
N.takasagoensis_NtSsp1_AB195158.1       ------------------------------------------------------------
R.aculabialis_RaSsp1_UES72773.1         ------------------------------------------------------------
R.flavipes__Deviate_HQ003932.1          ------------------------------------------------------------
D.melanogaster_NP_524497.1              ------------------------------------------------------------
```

```
                                           970       980       990      1000      1010      1020
                                    =========+=========+=========+=========+=========+=========+
RsTO1                               ------------------------------------------------------------
RsTO2                               ------------------------------------------------------------
RS001402                            ------------------------------------------------------------
RS001626                            ------------------------------------------------------------
RS001971                            ------------------------------------------------------------
RS002171                            ------------------------------------------------------------
RS002695                            ------------------------------------------------------------
RS004851                            ------------------------------------------------------------
RS007322                            ------------------------------------------------------------
RS007323                            ------------------------------------------------------------
RS010477                            ---------------------------RVVS---------------------------VD
RS013480                            ------------------------------------------------------------
RS013481                            ------------------------------------------------------------
RS013482                            -----------------------------------------------------------E
RS013500                            ------------------------------------------------------------
RS013501                            ------------------------------------------------------------
RS013565                            ------------------------------------------------------------
RS013761                            ------------------------------------------------------------
RS013762                            ----------------------------------------------------------FT
RS014379                            ------------------------------------------------------------
RS014493                            ------------------------------------------------------------
RS014811                            ------------------------------------------------------------
RS014812                            ----------------------------------------------------------LS
RS014813                            ------------------------------------------------------------
RS014814                            ------------------------------------------------------------
RS014823                            ------------------------------------------------------------
RS015583                            ------------------------------------------------------------
Z.nevadensis_XP_021917347.1         ------------------------------------------------------------
Z.nevadensis_XP_021924758.1         ------------------------------------------------------------
C.secundus_XP_023709343.1           ------------------------------------------------------------
C.secundus_XP_033607719.1           KLINANWRAVLKQLGESINEAFGLTIHRIFSEAAKTVPYKDFFDDIECSVTSQSHKREIE
C.secundus_takeout3_XP_023710423.1  ------------------------------------------------------------
C.secundus_takeout4_XP_023711845.1  -----------------------------------------------------------K
C.secundus_takeout9_XP_023709326.1  ------------------------------------------------------------
B.germanica_PSN55186.1              ------------------------------------------------------------
N.takasagoensis_NtSsp1_AB195158.1   ------------------------------------------------------------
R.aculabialis_RaSsp1_UES72773.1     ------------------------------------------------------------
R.flavipes__Deviate_HQ003932.1      ------------------------------------------------------------
D.melanogaster_NP_524497.1          ------------------------------------------------------------

                                          1030      1040      1050      1060      1070      1080
                                    =========+=========+=========+=========+=========+=========+
RsTO1                               MAEAR---------------------------------RTFV--FC-LILVSTETL----
RsTO2                               MDKCM---------------------------------KIIV--LC-LALFSADAL----
RS001402                            ------------------------------------------------------------
RS001626                            ------------------------------------------------------------
RS001971                            ------------------------------------------------------------
RS002171                            MLLLF-------------------------------------------------------
RS002695                            ------------------------------------------------------------
RS004851                            MKT----------------------------------QAILIAAVA-LLLGTEAGLIKPS
RS007322                            MKS-----------------------------------ATLLALLA-VVTLDSASI----
RS007323                            MKVGDSF-----------------------------FPVTLL--LA-VTIGNCDVN----
RS010477                            MRSVTVT-----------------------------SAAVLL--AANILLCHYSSSSHP-
RS013480                            MKAVH---------------------------------AVILA-LA-LFLGANSHAEAG-
RS013481                            MRFLQ---------------------------------AVILA-FS-LVLGVNSLPSADV
RS013482                            LQNIT---------------------------------SVLMT-MA-ILLGPFAGSEVTA
RS013500                            MGFLYLL-------------------------------SLLVL----LLVTSFGQICCSE
RS013501                            FKYLV---------------------------------CLLI--LL-VLVHCFGELES--
RS013565                            MKSLSIL-------------------------------VIFVA-LA-TLFHQSSSLETDI
RS013761                            LQFVL---------------------------------AALLA-TS-TLAAPSVSV----
RS013762                            MSAFS---------------------------------IFFV--AC-IVVFGSDGL----
RS014379                            MNATM---------------------------------YILCC----LLFVAGGNIVSSA
RS014493                            MKTVI---------------------------------------LL-VVLQLAIGA----
RS014811                            MSLVTDTACHMSLVSDTASHMSLPVTCLSADRHHTPGNGVTL--LS-YPVAASTGITCPG
RS014812                            MQNIF---------------------------------AVFL--AA-SAFTAGTAL----
RS014813                            ------------------------------------------------------------
RS014814                            ------------------------------------------------------------
RS014823                            LKSGA--------------------------------AAVLL-----LAAVTCGVV----
RS015583                            LSTPV-------------------------------ATSVLL--LGTMSLVRATTQ----
Z.nevadensis_XP_021917347.1         ------------------------------------------------------------
Z.nevadensis_XP_021924758.1         MNKYL---------------------------------VLLT--MC-LAASSTAAH----
C.secundus_XP_023709343.1           MERTH---------------------------------RIFV--LC-LVVFGCAAL----
C.secundus_XP_033607719.1           MEKTL---------------------------------ALLV--TC-CLLLSATAL----
```

```
C.secundus_takeout3_XP_023710423.1      MNAIV---------------------------------YILCC----LLIVAGGDTVSST
C.secundus_takeout4_XP_023711845.1      LQNII---------------------------------SVLMA-IA-ALLVSLADTDLRP
C.secundus_takeout9_XP_023709326.1      MELLF---------------------------------YMIL--LSPVLASGAQGPKRPQ
B.germanica_PSN55186.1                  --------------------------------------MFLR--LV-LLLLPVLGL----
N.takasagoensis_NtSsp1_AB195158.1       MQKCF---------------------------------AIVASCCC-LLLLCEAAL----
R.aculabialis_RaSsp1_UES72773.1         MYKCM---------------------------------KIIV--LC-LALSSADAL----
R.flavipes__Deviate_HQ003932.1          LQFVL---------------------------------AALLA-TS-TLAAPSVSV----
D.melanogaster_NP_524497.1              --------------------------------------AIAFA-VV-LCLLVSVDA----

                                              1090      1100      1110      1120      1130      1140
                                        =========+=========+=========+=========+=========+=========+
RsTO1                                   -------------------------------RLPDYIK----------------------
RsTO2                                   -------------------------------KLPDFVH----------------------
RS001402                                ------------------------------------------------------------
RS001626                                ------------------------------------------------------------
RS001971                                ------------------------------------------------------------
RS002171                                -------------------------------VTASFLK----------------------
RS002695                                ------------------------------------------------------------
RS004851                                PGT-------AGDLLQGLTRPA--------PNTRKVLP-------N-------------L
RS007322                                ------------------------------------------------------------
RS007323                                -------------------------------RLPESFL----------------------
RS010477                                ------------------------------AKLPKGFP----------------------
RS013480                                -------------------RINRQVLE---ARLRTFLAPGXRNE----------------
RS013481                                -----------------VRRIPRHQLE---AELRTLLAPGFKAA----------------
RS013482                                KPR----WI---DLISGITLTSRDVIVGTVERVREIFPPDKIVY----------------
RS013500                                AKSETHHWFVEGNKTSDALLTKRRVLEHD-TKDQDVFS--KIRG----------------
RS013501                                ------------------------------RNRNELFPTNWTDGD---------------
RS013565                                ----------------------RDEILKAYDKAKERLGPSESRI----------------
RS013761                                -------------------------------QWPSIFE----------------------
RS013762                                -------------------------------KLPDYIT----------------------
RS014379                                S-----------------------------EDTSEGAA----------------------
RS014493                                -------------------------------NLPDKFK----------------------
RS014811                                GQT--------------VTKTGKN------VSASAYFQ----------------------
RS014812                                -------------------------------QLPKYVT----------------------
RS014813                                ------------------------------------------------------------
RS014814                                ------------------------------------------------------------
RS014823                                -------------------------------QLPSYMK----------------------
RS015583                                -------------------------------ELPPYVK----------------------
Z.nevadensis_XP_021917347.1             ------------------------------------------------------------
Z.nevadensis_XP_021924758.1             -------------------------------KLPDYIK----------------------
C.secundus_XP_023709343.1               -------------------------------QLPDYMK----------------------
C.secundus_XP_033607719.1               -------------------------------KLPDFVK----------------------
C.secundus_takeout3_XP_023710423.1      S-----------------------------EDISEEVA----------------------
C.secundus_takeout4_XP_023711845.1      KSR----WM---DLISGITRTSKDVIMGTVGRVREIFPPEKIVY----------------
C.secundus_takeout9_XP_023709326.1      ------------------------------KPASAYFS----------------------
B.germanica_PSN55186.1                  -------------------------------ELPSYIK----------------------
N.takasagoensis_NtSsp1_AB195158.1       -------------------------------KLPDFVK----------------------
R.aculabialis_RaSsp1_UES72773.1         -------------------------------KLPDYVH----------------------
R.flavipes__Deviate_HQ003932.1          -------------------------------QWPSIFEPCDNSGPDFGDCAKHNLQTAVL
D.melanogaster_NP_524497.1              -------------------------------KFPEDPK----------------------

                                              1150      1160      1170      1180      1190      1200
                                        =========+=========+=========+=========+=========+=========+
RsTO1                                   --------PCSRNDPKFNDCALKHGRDAI--PRIVRGDRKLGVPVLDPLLVDKLSMTHG-
RsTO2                                   --------RCSVKDPEFDACVLKSAKETV--PHMIDGLKQYHIPVLDPLYVTEVRAVDG-
RS001402                                ------------------------------------------------------------
RS001626                                -------------------------------------DKERKIPFLDPLFIIELRVNDG-
RS001971                                ------------------------------------------------------------
RS002171                                --------VCHRSDPKLDECVMASVEGLR--SHLVTGIPELQIPSCEPLEIKQLVLNQGH
RS002695                                ------------------------------------------------------------
RS004851                                VLSPALAKALYYSQISLSSTIQRE-------AKLINKVPEKEL-VLDDLHIA--------
RS007322                                -------------------------------KAAKLRLPALHLLPVDPLDVTKITIEHGA
RS007323                                --------RCRRHDPDLNSCLQAAIQKAI--LLMKDGIPELQLLPVDPLEVTNISIQDGV
RS010477                                --------RCSRNQPELNKCLGPAIRTAL--TVMKDGIPSHHELPIDPLLVTSISLNIGA
RS013480                                ----------------LDDAVKRLLETKV-RDIIKNGDEELGLPVLDPLXVEHLDINLNQ
RS013481                                ----------------LDDVIANLLDTTV-RQIILEGSEDLGIPVLDPLKIDHLDLDINL
RS013482                                ----------------PDDFVISEIVQYF-KGKMKYPHPSLELPILDPFFQRQLDFNITH
RS013500                                ----------------KERVFEEQLEGLI-RAILENGIPDLNIPPLDPLVYKDIVYISET
RS013501                                --------SLSQGEAYVDNIF----------NKILSNNGAKKGAMIQPFPLEELYFHDNE
RS013565                                ----------------LEDIILGNII-----SAIKNGSECFGNPPLDPLDYQENIHLENV
RS013761                                --------PCATSRPDFGDCAKQNLQTAV--RNFSAGVPELDIPPFDPIFVPVVLLDYKR
RS013762                                --------PCSRKDPNFNKCALKHGREAI--PKFLKGDPKYGVPNLDPIFVKEISIRES-
RS014379                                ----GLTNVTLLGEQKLSDQVRLILNHYK--EDDPVGLP--GAPISDPMPIPDMKHSFTY
RS014493                                --------RCHVNEPEGSSCLKVAVADAL-RKIGTTGIPSLNVDPLQPMTVKEVKVDQGG
RS014811                                --------ICHKSDPDISTCVKNTIEDMR--PKLITGIPELELVPLDPLVIPRLEFNEGS
```

```
RS014812                             --------PCARSDTNFNDCVKEHAQETLASPILEKGDRKYNIPALSPLLIKELDVSEG-
RS014813                             ---------------------------------------KYKVPVLEPLDIAELKIADSG
RS014814                             -------------------------------------DPKYRVPPLDPLLIEELSVKQGS
RS014823                             --------PCARSDPKFNECALEHAKETF--PQFVKGDRKYKIPRMDPLDIKEMIVSQGA
RS015583                             --------QCPEGDPKIVECITGALHHLR--PYLAKGIPEIEMPSVEPFRMDELSLSLTT
Z.nevadensis_XP_021917347.1          ----------------------------------------------------------M-
Z.nevadensis_XP_021924758.1          --------KCSLKDPEFNACALKSARAAI--PHLVKGAKKHNIPLLDPLIVKEVRAVES-
C.secundus_XP_023709343.1            --------PCSRSDPNFNACALQQGREAM--SRMVQGDDKYGVPVLDPLLIRKLTTTYN-
C.secundus_XP_033607719.1            --------KCSLKDPEFNACALKNAREAL--PHIVNGLKKFQIPVLDPLIVEEIRAVDG-
C.secundus_takeout3_XP_023710423.1   ----ALSNVTIFGEQKLSDQVRMILDHYK--QEDPVGLP--GAPVSDPMPIPDMKHSFTY
C.secundus_takeout4_XP_023711845.1   ----------------PDDSIISEFVEYL-KGKMKYPHPSLELPVLDPFVLKQLDFNITN
C.secundus_takeout9_XP_023709326.1   --------ICHRSDPNISGCIKNTIEDLR--PTLIKGTPELDLIPLDPLNIPRLELREGG
B.germanica_PSN55186.1               --------PCPRNDPNLDECIFKNGQEAV--PFIIKGDPEYGLFVMDPWHPKDLRIKEL-
N.takasagoensis_NtSsp1_AB195158.1    --------KCSAKDPNFDDCVFEHAKETI--PHMIDGLKKYRIPVLDPIHVKEMRANVG-
R.aculabialis_RaSsp1_UES72773.1      --------RCSVKDPEFDACVLKSAKETV--PHMIDGLKQYHIPVLDPLYVTEVRTVDG-
R.flavipes__Deviate_HQ003932.1       NFSAGIFKPCATSRPDFGDCAKQNLQTAV--LNFSAGVPELDIPPFDPIFVPVVLLDYKR
D.melanogaster_NP_524497.1           --------PCKYGD---GECIMKLCNTLF-SENSAEGDPGLNLMQLDPLKVDRMVISQGE

                                           1210      1220      1230      1240      1250      1260
                                     =========+=========+=========+=========+=========+=========+
RsTO1                                -----GIH---ATSKNFTIVGVKDAVLEYFS---ADFDKHVIKLTFSSPRVVLSGD-YEV
RsTO2                                -----DLN---MAALNVKVEGIRNIIIKSAR---FDLDKKEITVETVVPEAHFSGN-YEV
RS001402                             ------------------------------------------------------------
RS001626                             -----KLN---GAILNTNVTGLKDLALDSVR---FDFDNKNITVEGRLPALNFXGN-YEV
RS001971                             ------------------------------------------------------------
RS002171                             G--AVSLT---STYRDIKLYGPTGFRLDDVR---IDLDNKRVHIQLWLPFLRMTSH-YKI
RS002695                             ------------------------------------------------------------
RS004851                             --IDDDIS---VQLSNLKLLNVPSIDVTSAN---ANLEFLNLALDIDFGDLQVAGN-FEV
RS007322                             G-RPVSLN---LEFNNVKSAGLSQSEVKTVR---IDLDKRILEADAFVPKTVMEAD-YVM
RS007323                             G-RPVKMS---LDMNKAKLHGLTQCRLKAVR---ADLEKRCIELEANVPFTTIDAD-YIM
RS010477                             D-QSVNFK---LDMTNVSLIGLNDVDVLSAS---LDMKEHRYDVQVHGPKFELKGD-YEI
RS013480                             D-------------------------------------------GLSLDAARLEGEHYKL
RS013481                             D-TTAQLK---GQLDGVEVRKIATFVVDNIK---ANLLLLKVDFAISVPEIVAEGEHYKL
RS013482                             D-MIGQLS---LYLEELNITKMSEFNVNKMK---MNWLRRKLEFDIRFPEIHIRTK-YKM
RS013500                             D-MPGMMTIEFLELGNFGVFGLSKFINHLMV---FDLTQLAITLNFTFP-IVVEAN-HSN
RS013501                             R----EFS---MHLHNMQGSSKFVFNIKKLQ---TILKERHVELELLMPNLFVEGS-YEC
RS013565                             DLLNYVLV-DFLNVDGVRSTKLSEFLVHTFI---FDLTGLAVVLNVTFPEINLDLDHYDL
RS013761                             E--NAEGK---MIIRNTKVYGLKDGEILDFRANLSDPSNLLIEVDFRLPSVFVEGQ-YKA
RS013762                             -----GLT---IIARDMTMEGAKNAVLQDIS---IDFDKLTISLQFLVPLAVFTGK-YDM
RS014379                             A---------TMHFKSAHVHGLSRFRIQHIK---SNLAAMQVSVGLRIEKLEVLGN-YTM
RS014493                             K-GPVAIK---LQFTNLKIYGIPESSILSFGL------TNHVVCHVTDSLITY----HDI
RS014811                             G--NFRFS---QVLTNVTIRGLGAFRLRNV------------------------------
RS014812                             ----TGLK---IKMRDVKIFGVLGASVEKLN---TDFDKQQIDGLLRFPAVSIIGK-YEA
RS014813                             P-RQAGLT---LVMTNAKVYGVKDSYLENTD---FDFDKQHVHHEMTLRRLEILGK-YNV
RS014814                             P--NFGLS---FTARNVSIRGLKNVQVKAVR---IDLQKQHIEYDLLVPTVEVLCR-YSV
RS014823                             S--QTGFS---LTMRDVKMYGLKDALIKKTD---FDFDKNHIIYDTTLPLLTILAK-YEI
RS015583                             G--PNGYK---VTLRDLDIYGASNFTISDLK---LSHGDAPFEAHIKIPELKINAR-YTS
Z.nevadensis_XP_021917347.1          -----GLN---ASSSNFTIVGVKDAFFEDIS---IDFEKQIIRVSFLIPHAVLNGD-YSI
Z.nevadensis_XP_021924758.1          -----YLT---MAAIDVKMQGLKDAVLQNIS---FDFDKQNITAEVLVPEAHFTGN-YEL
C.secundus_XP_023709343.1            -----GFT---ATTRNSTVVGVKGVVIEDIS---FDFDKQLVRVAFMFPTLQFNGL-YEM
C.secundus_XP_033607719.1            -----QID---MAGLNVRVEGIRNAILESIS---FDFDQKKIAAEVVVPEAHFSGR-YEV
C.secundus_takeout3_XP_023710423.1   A---------TMHFKSAHVHGLSRFRIQHIK---SDLAALKVSVGLRIEKLEVLGN-YTM
C.secundus_takeout4_XP_023711845.1   E-MIGEFT---LNLEELRIIMMSEFDINKIK---MNWLPRKLEFSVRFPEIHTQAK-YNM
C.secundus_takeout9_XP_023709326.1   G--NFRFE---QVLTNVTIHGLGAFKLLNVK---ADTDNLTLDMHMLTPFMRFDAY-YEM
B.germanica_PSN55186.1               -----GLS---LVGFNMSIVGYKGGHLDSVK---TDPSKNKVTIKYRVPEILFTGK-YDM
N.takasagoensis_NtSsp1_AB195158.1    -----GLD---MHGWDLVGTGLRNVQLKNIK---IDLKKKEITVEVLVPKAHFQGK-YEV
R.aculabialis_RaSsp1_UES72773.1      -----DLD---MGAQNVTAEGIRNIIIKSVR---FDLKKKEITVETVVPEAHFTGN-YEV
R.flavipes__Deviate_HQ003932.1       E--NAEGK---MIVRNTKVYGLKDGEILDFRANLSDPSNLLIEVDFRLPSVFVEGQ-YKA
D.melanogaster_NP_524497.1           SSSPVGIT---LTFTDNLLYGIKDQRIVKVKGFGRDLTAKH-EVKIVTKTFSLVGP-YNI

                                           1270      1280      1290      1300      1310      1320
                                     =========+=========+=========+=========+=========+=========+
RsTO1                                GGNIM--GLPINGQGDYELFFDGLRGNYTTNYT-LTQLEDGELYAVPDTYDAEFE-TQGM
RsTO2                                KGKLL--ALPIVGKGPLDSKFYDLYVKYVTQYE-LKKLDDGEEHLMPYNYTVDFE-PRHV
RS001402                             ----------------------NMTTSVSMLFE-LPRL-QGRQVIHIVDMKVDFD-IQGM
RS001626                             SGKLV--GHNISGNGKFNATLHDNSVKYTTSYD-LNTHDDGQVYLVLQSYHLDLE-PGKL
RS001971                             --------------------------------------------------------MGNM
RS002171                             AGRVL--ILPIAGSGYSEGNYTDINATCSLQGE-HIDI-NGRTHFSVKYFDVKFS-IGDA
RS002695                             ----------------------NLTILYKATYD-LRKMDDGQVYMIFTXYDCHIX-PEHV
RS004851                             VSKKTLAEIPVTSSGEFVLRNSPFKGTGKVGLS------LQTNFYKTINYDVVYQ-PTGS
RS007322                             DGRFL--VLPVKGNGKCKFDFTGMNATVKIEAE-PQTK-SGKVYWNVVNLEIDIKSMENF
RS007323                             DGKFL--VLPMKGKGKCNMNLTDYDVSVKIRAE-PMVK-NEMVYWDVKEFHLRIDSLKLF
RS010477                             HGRFL--LLPLTGKGKFEMAFLSTDVDMHIPYE-EKTK-GGDVYWHIKPYNILMKNTKKL
RS013480                             EGDILNGALPVEGEGRFEADLKRLGLKVKLELG-V----KAEGDLEVRKLELDVS-LGEG
RS013481                             DGNLG-GLLPVYGEGRFAASVKNISLTGSVTLG------TNNSFIYVKSLVLDLN-LSEA
```

```
RS013482                              KGILG-NVLPVHGNGPASLSAINVNFTGTIDLD------THNNSLRIKTLLLKYY-IEQF
RS013500                              FSIIVGDLIPFNGEGKANL-IADLRIEAYIGLG----R-TVDTYLYLDALSADIF-FDSL
RS013501                              DRMAY-GDYESKSSGNFRLTLNNLTAEGLASLK-I----ENNSSLKVDKMRLMYS-STYK
RS013565                              SAVAL-TIIPVDGNGTLQLTLRDVNVDLAFTLG------SQNGSIFLDSLSLHLA-VGEV
RS013761                              QGKIV--GFPLGGKGVYNISLSEVTGTWGVQGE-LVTI-AGDQYLQVRHVNILPE-VGDM
RS013762                              SGKLA--NLPISGKGDFKVSFVESTVKYKTFCN-LTQHSDGKTYVQPYEYDLAFD-PKTG
RS014379                              SSWFS------RSNGPFNVTLSNVYIEGLAKLE-V----ERGGQLQAQNIDMDIT-FQDI
RS014493                              RGK-------------------------------PVTR-KGDTYFDLQTYEIKMR-TKKM
RS014811                              ------------------------------------------------------------
RS014812                              AGRIL--VLPLTGNGNINITLVNVTLPADLDFS-VQPI-GSTKHFVVKDVRATVL-PSRV
RS014813                              SGRLL--LVPLTGNGDINITLENVQITYDHDYT-LEPI-KGDIYMVIGNTSSTLE-PSMA
RS014814                              SGQVL--VVPISGSGNGVLVFKDLDLRIAFDFDKVRKKPQGKEYVSPKNFLITTE-AHGL
RS014823                              GGRIL--ILPISGKGDINITMINNRLTYSYDFT-IQER-DGVKYHASKNERAVVV-PSDG
RS015583                              SGVLI--ILPASGQGNFHSTLGDIIATVQGTIS-SQQR-AGRDYLHVDDLDINLN-IKTV
Z.nevadensis_XP_021917347.1           AGNLV--GLPIYGKGLYELSFDGLSANYSTNYS-LTTLQDGELYVIPQAYDFDFD-VKLM
Z.nevadensis_XP_021924758.1           DGRLV--ALPITGKGPLDATLYDLYVKYTTGYS-FTKMDGGEVYLVPTDYXIYSQ-PKNL
C.secundus_XP_023709343.1             SGKLA--GLPISGKGDYDLFLSGLSGNYSTNYT-LARLRDGQLHAMPQSYAVDFD-IRGL
C.secundus_XP_033607719.1             KGKLL--TLPLSGNGPFNSTFYHLYVKYVTTFD-LTKHDDGHVYLEPKEYKVHFQ-PKNV
C.secundus_takeout3_XP_023710423.1    SSWFS------RSSGPFNVTLSDVYIEGLAKME-V----ERGGQLQAQNIDMDIA-FRDI
C.secundus_takeout4_XP_023711845.1    KGILG-NLVPVHGHGPASISAINVNLTGMVDLG------IHNNSLHIRKLTLNYF-IEAF
C.secundus_takeout9_XP_023709326.1    EGKVL--VVPLSGKGDCVVNFTDVTTIAHTQLE-LVTR-DGDQYLNVQKVEWNID-AENC
B.germanica_PSN55186.1                NGRLV--AIPVRGNGDFVVNMYDTVIKYTTEFK-VVRKGDGEEYAVPQSYEFLYA-PGDM
N.takasagoensis_NtSsp1_AB195158.1     NGKIM--SLPITGKGDFEATFDDLYAKYVTTYE-LQKMDDEEVYLVPTIYNVIFE-TKSA
R.aculabialis_RaSsp1_UES72773.1       KGKLM--SLPIVGKGPLDAKFYDLYVKYVTNYE-LKKLDDGEVHLMPYNYTGEFE-PRRV
R.flavipes__Deviate_HQ003932.1        QGKIV--GFPLGGKGVYNISLSEVTGTWGVQGE-LVTI-AGDQYLQVRHVNILPE-VGDM
D.melanogaster_NP_524497.1            QGKVL--ILPISGTGQSNMTMVNVRAIVSFSGK-PLVK-NGETYLDVTDLKITMK-PESS

                                            1330      1340      1350      1360      1370      1380
                                      =========+=========+=========+=========+=========+=========+
RsTO1                                 KINFRNLFN--GNKLMGDAMNNFINDNWRIVLEDIGKPIFVALGSIVHQILMNVSQ----
RsTO2                                 TATLGNLFN--GNKVLGENMNKLINENWREVLKVVGKPTYDALGLVVHTIVSGASK----
RS001402                              TVQFDNLFN--GNEVLGRTLNAFVNRNALDVVNELKEPLGESFSLVFKDIMNNAFS----
RS001626                              TAQLDNLFN--GINLLGDEMNRFINDNWREVMDQVGRDVYDAFGLVIHKIFKESAQ----
RS001971                              ELHITNLLQ--GRAVMESILDGIINVSWRPFLPIVKPLIDDLVSTAFTDIFNNNFQ----
RS002171                              QLFLRDLFN--GDRDLGDAMNLFLNRNWRNVATDLQPLLESKIGELLKKFSNNIYH----
RS002695                              QXRXEXLFN--GNKLLGEAMNTFLNENWRLVYTEFGKSLSLAVGEFLFNIIKESVK----
RS004851                              YLTVSYLDN--NNKRIISGLEA--KNQEAKVVAHINRQLKNLLNRLVQKRLDDILA----
RS007322                              RVKFDNLFN--GDKALGDSTNKVLNDNWKQFWEELKPSFEETFAAVFLQLAKAVFS----
RS007323                              QVNFENLFN--GDKELGDNTNKVLNENWKEFFEELRPILRDTFGAEFLQYTNQLFH----
RS010477                              TVHFDHLFN--GDKMLGESTNSVLNENWEVLWNELRPRFQDTFGQIFAEIANTVYS----
RS013480                              KVNFENLLG--GGT-LGEIANDFISSELPGLVDSNKGPILLTIADEIRRIANEKLV----
RS013481                              EVNLEGILG--GGD-LADLVNQIVSNLIPDLLEELKPTLLPDITQGIIDLANEKLD----
RS013482                              QINMMNLMG--DKR-LGELLNTVLSEEGTQILELVRTDVTHFGTNWLHSVGNDVLGK---
RS013500                              EVEFSTLMGVTGGK-ETHVFNKLIGDVTPELIDIMKPYMIDGILESLLTSVNEFLLP---
RS013501                              RIQVRQQET--NPR-LSTFITSV--AFGKMVVRKIWRSLTYNLNTVIEGKVNLALR----
RS013565                              KLTVTGLFG--GSR-TSYLAGAIVSSLAPAAIEDIQASNGPEIVEELKALINGLLVG---
RS013761                              KIYASNLFT--GNEEISE------------------------------------------
RS013762                              EIYFGNLFN--GNKLLGDTMNNFLNENFRLVFEEVAQPAYKALGSIVDQILRGVFE----
RS014379                              AMNFQNLGF------LGNLFQGIINSVGTFLFDSIKPFILSEVNTNVRGDVNKHIRALPQ
RS014493                              NLNFRNLFN--GDKALGEAMNKVINENWELIFKELRPALEKAFGDVFLEYAKGILD----
RS014811                              ------------------------------------------------------------
RS014812                              YIHLTNLLG--NDTLLEKAMNDFLNNNWKEVFDIMAPSITAAIVKVLGNMFNGIAA----
RS014813                              HIRLTNLFN--GDPLLGNQMNLFLKENWREVYKELSPAVITAFTEIVNSIVKGIAG----
RS014814                              TVNLENLFN--GNKFLGDNMNTFLNENWRDLMHELAPPVGEALIQVLETTLTNIFE----
RS014823                              RISLTNLFN--GDKQLGNQMNNFLNDNWKDIYKTMSPAISEAFAQVIGNIVNNIAD----
RS015583                              RMVVKKVFN--NNRILTEATNLFLRENGHEVLKAMMPQLRHKLSEVFKGISNQLLT----
Z.nevadensis_XP_021917347.1           KVHVENLFN--GNKFLGDNMNTFLNENWRLVIDDLGKPMSHALGSIVHRILSNIYK----
Z.nevadensis_XP_021924758.1           KAKLGNLFN--GNKLLGDSMNAFINENWQAVLKEVGQPTYDALGLIVHTILSAAAK----
C.secundus_XP_023709343.1             KVHFGNLFK--GNRLLGNSINDFINENWRLVVEELGKPVLTSLGSIVHEVLLNLAE----
C.secundus_XP_033607719.1             KAHLGNLFN--GNTVLGDIMNQFINENWREVLQELGQPTYDALGLVVHKILNNTMR----
C.secundus_takeout3_XP_023710423.1    AMNFENLGF------LGNLFQGIINSVGTFLFDSIKPFILREVNTNIRGDVNKHIRALPQ
C.secundus_takeout4_XP_023711845.1    RINMTNLMG--GGR-ISELMNMVLSQEGTEILELVRTDVTYVSTNWLHIVANDVLQK---
C.secundus_takeout9_XP_023709326.1    HFHFNNLFG--GDKALGANTNAFLNKNWREAFDTFKYLAEEAFGILFRDLTNRVYR----
B.germanica_PSN55186.1                KMNLTNLFG--GNKLIGDVMNKFMNDNWRAVMEEAGKPSSRAVGQEVHQLTSIIVQ----
N.takasagoensis_NtSsp1_AB195158.1     NVRMDNLFN--GNKLLGDNMNKFLNENWQDVMKEAGQSTYNALGLVIHNIFSGTTL----
R.aculabialis_RaSsp1_UES72773.1       TAKLGNLFN--GNKVLGDNMNTFINENWREVLKVVGKPTYDALGLIIHTMITEASK----
R.flavipes__Deviate_HQ003932.1        KIYASNLFT--GNEELNNAALQFVNTYWPAFYKEMLPFAAKGWDEFLSGVLNKLFL----
D.melanogaster_NP_524497.1            HYHFSNLFN--GDKALGDNMNVFLNENSEAIYKETAKAIDRSFGKLYLGVVKGVFS----

                                            1390      1400      1410      1420      1430      1440
                                      =========+=========+=========+=========+=========+=========+
RsTO1                                 -----KVPYNELFTN---------------------------------------------
RsTO2                                 -----TVPYKNIFLD--------------VE-----------------------------
RS001402                              -----HMPIDIWLLD--------------K------------------------------
RS001626                              -----TVPYKDIFND--------------TE-----------------------------
```

```
RS001971                                  -----NFPFRELFPR---------------------------------------------
RS002171                                  -----KFTLDQLLPP---------------------------------------------
RS002695                                  -----TVPFNEIFND--------------VE-----------------------------
RS004851                                  -----GVPVDKLIGETRTAESYREHARALRVVANEYADQLLENVRSYVVENGLSEVAVPN
RS007322                                  -----RVPEENIFLE---------------------------------------------
RS007323                                  -----KIPENELFLE--------------S------------------------------
RS010477                                  -----RVPIKDHFLD---------------------------------------------
RS013480                                  -----GVTLDDLLKL---------------------------------------------
RS013481                                  -----GVTLQDILDL---------------------------------------------
RS013482                                  ----MSVSLSGVLDF---------------------------------------------
RS013500                                  ----LKITYTDIINC---------------------------------------------
RS013501                                  -----IRPLTQIIGNSQTLTKYEEYVNTTTESANKLVDVIVEYAKDLILQKYSDRIKIPD
RS013565                                  ----DGESSTNIISC-------------------------------LIM-----------
RS013761                                  ------------------------------------------------------------
RS013762                                  -----KVPYDELFTD---------------------------------------------
RS014379                                  RFPNSISPLDMALAE----------------------------GRRHVREKGYDPHHFPD
RS014493                                  -----KVPFSNIFLK---------------------------------------------
RS014811                                  ------------------------------------------------------------
RS014812                                  -----VVPFDEVFPE--------------TLP----------------------------
RS014813                                  -----AVPFDVAFPE--------------KLPAD--------------------------
RS014814                                  -----LVSYDDSFPE--------------TV-----------------------------
RS014823                                  -----VLPFDVLFPE--------------TIP----------------------------
RS015583                                  -----HVSTDMLLQPAKA------------------------------------------
Z.nevadensis_XP_021917347.1               -----KIPFKEMFAE---------------------------------------------
Z.nevadensis_XP_021924758.1               -----TVPYKDIFDD--------------TE-----------------------------
C.secundus_XP_023709343.1                 -----KIPYDELFAA---------------------------------------------
C.secundus_XP_033607719.1                 -----VVPYKDLFDD--------------TD-----------------------------
C.secundus_takeout3_XP_023710423.1        RFPNSISPFDMALAE----------------------------GRRYVREMGYDPHLFPD
C.secundus_takeout4_XP_023711845.1        ----MSISFSGLLDF---------------------------------------------
C.secundus_takeout9_XP_023709326.1        -----HFTYKELLPE---------------------------------------------
B.germanica_PSN55186.1                    -----QL-----------------------------------------------------
N.takasagoensis_NtSsp1_AB195158.1         -----TVAYKDVFDD--------------VEV----------------------------
R.aculabialis_RaSsp1_UES72773.1           -----TVPYKNVFLD--------------VE-----------------------------
R.flavipes__Deviate_HQ003932.1            -----QVPFNTIFPA------------------N--------------------------
D.melanogaster_NP_524497.1                -----KLPYAKFFAD--------------ES-----------------------------

                                                1450      1460      1470      1480      1490      1500
                                          =========+=========+=========+=========+=========+=========+
RsTO1                                     ------------------------------------------------------------
RsTO2                                     ------------------------------------------------------------
RS001402                                  ------------------------------------------------------------
RS001626                                  ------------------------------------------------------------
RS001971                                  ------------------------------------------------------------
RS002171                                  ------------------------------------------------------------
RS002695                                  ------------------------------------------------------------
RS004851                                  IEQ--SFSKEILLITWHGSFATRDGHARNLASLVRRGDFNLDVDASTGAIIVS-GSLGLT
RS007322                                  ------------------------------------------------------------
RS007323                                  ------------------------------------------------------------
RS010477                                  ------------------------------------------------------------
RS013480                                  --------------------------------INNSK-----------------------
RS013481                                  --------------------------------INGGGLKKL-------------------
RS013482                                  --------------------------------MQTAGTQKVR------------------
RS013500                                  --------------------------------LMGSDKCPFDL-----------------
RS013501                                  IRE--GFEKKVLFIAWKGHFLAHSGTARGCHTVERVGDTTFANDNSS-FMHLY-GTLGFE
RS013565                                  ------------------------------------------------------------
RS013761                                  ------------------------------------------------------------
RS013762                                  ------------------------------------------------------------
RS014379                                  YNYTVGIFRVDLTHSW----------VSGISSFYRVGNISVTMEDNIVYVGVHVGTQRLE
RS014493                                  ------------------------------------------------------------
RS014811                                  ------------------------------------------------------------
RS014812                                  ------------------------------------------------------------
RS014813                                  ------------------------------------------------------------
RS014814                                  ------------------------------------------------------------
RS014823                                  ------------------------------------------------------------
RS015583                                  ------------------------------------------------------------
Z.nevadensis_XP_021917347.1               ------------------------------------------------------------
Z.nevadensis_XP_021924758.1               ------------------------------------------------------------
C.secundus_XP_023709343.1                 ------------------------------------------------------------
C.secundus_XP_033607719.1                 ------------------------------------------------------------
C.secundus_takeout3_XP_023710423.1        YNYTVGIFRIDLTNSW----------ASGISSFYRVGNVTVTMEDNVLYLGVHVGTQRLE
C.secundus_takeout4_XP_023711845.1        --------------------------------IKSAGTKKVK------------------
C.secundus_takeout9_XP_023709326.1        ------------------------------------------------------------
B.germanica_PSN55186.1                    ------------------------------------------------------------
N.takasagoensis_NtSsp1_AB195158.1         ------------------------------------------------------------
R.aculabialis_RaSsp1_UES72773.1           ------------------------------------------------------------
```

```
R.flavipes__Deviate_HQ003932.1          ------------------------------------------------------------
D.melanogaster_NP_524497.1              ------------------------------------------------------------

                                              1510      1520      1530      1540      1550      1560
                                        =========+=========+=========+=========+=========+=========+
RsTO1                                   ------------------------------------------------------------
RsTO2                                   ------------------------------------------------------------
RS001402                                ------------------------------------------------------------
RS001626                                ------------------------------------------------------------
RS001971                                ------------------------------------------------------------
RS002171                                ------------------------------------------------------------
RS002695                                ------------------------------------------------------------
RS004851                                ELSIHYNHYEVEFQDL-GPSGSLSADVGTNSIFLKISLVLAPEISITLEDFRIEEATDIS
RS007322                                ------------------------------------------------------------
RS007323                                ------------------------------------------------------------
RS010477                                ------------------------------------------------------------
RS013480                                ------------------------------------------------------------
RS013481                                ------------------------------------------------------------
RS013482                                -----------EILNL-YPFKHKF------------------------------------
RS013500                                ------------------P-----------------------------------------
RS013501                                HIDLFYDHYEAHFMGL-GPSGSLSTKFFPLDLYVHIHIDFT-GKKVYLDDFKVMSVGNLE
RS013565                                ------------------------------------------------------------
RS013761                                ------------------------------------------------------------
RS013762                                ------------------------------------------------------------
RS014379                                GRTL----WEVNVGGLLSRAGSATFTVEYIHTKTEISQPLDTRKRPVLRDLDI-DVGNIQ
RS014493                                ------------------------------------------------------------
RS014811                                ------------------------------------------------------------
RS014812                                ------------------------------------------------------------
RS014813                                ------------------------------------------------------------
RS014814                                ------------------------------------------------------------
RS014823                                ------------------------------------------------------------
RS015583                                ------------------------------------------------------------
Z.nevadensis_XP_021917347.1             ------------------------------------------------------------
Z.nevadensis_XP_021924758.1             ------------------------------------------------------------
C.secundus_XP_023709343.1               ------------------------------------------------------------
C.secundus_XP_033607719.1               ------------------------------------------------------------
C.secundus_takeout3_XP_023710423.1      GKTL----WELNVGGLLSRAGSSSFTVEYIHTEIEISQPLDTRKRPVLRDLDI-KVGNIQ
C.secundus_takeout4_XP_023711845.1      -----------EIFNL-YPFKSEAKSCT--------------------------------
C.secundus_takeout9_XP_023709326.1      ------------------------------------------------------------
B.germanica_PSN55186.1                  ------------------------------------------------------------
N.takasagoensis_NtSsp1_AB195158.1       ------------------------------------------------------------
R.aculabialis_RaSsp1_UES72773.1         ------------------------------------------------------------
R.flavipes__Deviate_HQ003932.1          ------------------------------------------------------------
D.melanogaster_NP_524497.1              ------------------------------------------------------------

                                              1570      1580      1590      1600      1610      1620
                                        =========+=========+=========+=========+=========+=========+
RsTO1                                   ------------------------------------------------------------
RsTO2                                   ------------------------------------------------------------
RS001402                                ------------------------------------------------------------
RS001626                                ------------------------------------------------------------
RS001971                                ------------------------------------------------------------
RS002171                                ------------------------------------------------------------
RS002695                                ------------------------------------------------------------
RS004851                                VEVTGLGIL-DWLVSHITTWVIDLFHDKIVGALEGQLRDYINAVLPSVTPDVFFK-----
RS007322                                ------------------------------------------------------------
RS007323                                ------------------------------------------------------------
RS010477                                ------------------------------------------------------------
RS013480                                ------------------------------------------------------------
RS013481                                ------------------------------------------------------------
RS013482                                ------------------------------------------------------------
RS013500                                ------------------------------------------------------------
RS013501                                VKLMGYGWLFDFLTSKVTTWIVGLYRDKIITDIQYKYDHYISGLLDTYDLDDILEGKLLR
RS013565                                ------------------------------------------------------------
RS013761                                ------------------------------------------------------------
RS013762                                ------------------------------------------------------------
RS014379                                VRMDGAGTL-DYVIEFVVNVLPNLLRYQIVNAVEGPIKLRVQDVMDSIDIEKMIQEKLPE
RS014493                                ------------------------------------------------------------
RS014811                                ------------------------------------------------------------
RS014812                                ------------------------------------------------------------
RS014813                                ------------------------------------------------------------
RS014814                                ------------------------------------------------------------
RS014823                                ------------------------------------------------------------
RS015583                                ------------------------------------------------------------
Z.nevadensis_XP_021917347.1             ------------------------------------------------------------
```

```
Z.nevadensis_XP_021924758.1          ------------------------------------------------------------
C.secundus_XP_023709343.1            ------------------------------------------------------------
C.secundus_XP_033607719.1            ------------------------------------------------------------
C.secundus_takeout3_XP_023710423.1   VRMDGAGTL-DYVIEFVVNVLPNLLRYQIVNAIEGPLKTRLQDIMDSIDVEEVLEEKLPQ
C.secundus_takeout4_XP_023711845.1   ------------------------------------------------------------
C.secundus_takeout9_XP_023709326.1   ------------------------------------------------------------
B.germanica_PSN55186.1               ------------------------------------------------------------
N.takasagoensis_NtSsp1_AB195158.1    ------------------------------------------------------------
R.aculabialis_RaSsp1_UES72773.1      ------------------------------------------------------------
R.flavipes__Deviate_HQ003932.1       ------------------------------------------------------------
D.melanogaster_NP_524497.1           ------------------------------------------------------------


=                                     =====
RsTO1                                ------
RsTO2                                ------
RS001402                             ------
RS001626                             ------
RS001971                             ------
RS002171                             ------
RS002695                             ------
RS004851                             ------
RS007322                             ------
RS007323                             ------
RS010477                             ------
RS013480                             ------
RS013481                             ------
RS013482                             ------
RS013500                             ------
RS013501                             LRA---
RS013565                             ------
RS013761                             ------
RS013762                             ------
RS014379                             LDKIKL
RS014493                             ------
RS014811                             ------
RS014812                             ------
RS014813                             ------
RS014814                             ------
RS014823                             ------
RS015583                             ------
Z.nevadensis_XP_021917347.1          ------
Z.nevadensis_XP_021924758.1          ------
C.secundus_XP_023709343.1            ------
C.secundus_XP_033607719.1            ------
C.secundus_takeout3_XP_023710423.1   LDKLKL
C.secundus_takeout4_XP_023711845.1   ------
C.secundus_takeout9_XP_023709326.1   ------
B.germanica_PSN55186.1               ------
N.takasagoensis_NtSsp1_AB195158.1    ------
R.aculabialis_RaSsp1_UES72773.1      ------
R.flavipes__Deviate_HQ003932.1       ------
D.melanogaster_NP_524497.1           ------
```
